# The first A-to-I RNA editome of hemipteran species *Coridius chinensis* reveals overrepresented recoding and prevalent intron editing in early-diverging insects

**DOI:** 10.1007/s00018-024-05175-6

**Published:** 2024-03-13

**Authors:** Yuange Duan, Ling Ma, Jiyao Liu, Xinzhi Liu, Fan Song, Li Tian, Wanzhi Cai, Hu Li

**Affiliations:** Department of Entomology and MOA Key Lab of Pest Monitoring and Green Management, College of Plant Protection, China Agricultural University, Beijing, 100193 China

**Keywords:** A-to-I RNA editing, *Coridius chinensis*, Hemiptera, Recoding, Evolution

## Abstract

**Background:**

Metazoan adenosine-to-inosine (A-to-I) RNA editing resembles A-to-G mutation and increases proteomic diversity in a temporal-spatial manner, allowing organisms adapting to changeable environment. The RNA editomes in many major animal clades remain unexplored, hampering the understanding on the evolution and adaptation of this essential post-transcriptional modification.

**Methods:**

We assembled the chromosome-level genome of *Coridius chinensis* belonging to Hemiptera, the fifth largest insect order where RNA editing has not been studied yet. We generated ten head RNA-Seq libraries with DNA-Seq from the matched individuals.

**Results:**

We identified thousands of high-confidence RNA editing sites in *C. chinensis*. Overrepresentation of nonsynonymous editing was observed, but conserved recoding across different orders was very rare. Under cold stress, the global editing efficiency was down-regulated and the general transcriptional processes were shut down. Nevertheless, we found an interesting site with “conserved editing but non-conserved recoding” in potassium channel *Shab* which was significantly up-regulated in cold, serving as a candidate functional site in response to temperature stress.

**Conclusions:**

RNA editing in *C. chinensis* largely recodes the proteome. The first RNA editome in Hemiptera indicates independent origin of beneficial recoding during insect evolution, which advances our understanding on the evolution, conservation, and adaptation of RNA editing.

**Supplementary Information:**

The online version contains supplementary material available at 10.1007/s00018-024-05175-6.

## Introduction

### A-to-I RNA editing in metazoans and the multiple origins of extensive recoding

RNA editing is prevalent in all domains of lives ranging from bacteria [1, 2], fungi [3–7], plants [8–11] and animals [12–17]. In metazoans, adenosine-to-inosine (A-to-I) RNA editing catalyzed by ADARs is the most abundant editing type [18–20]. Since inosine is read as guanosine (G), A-to-I RNA editing is able to recode the coding sequence (CDS) and lead to nonsynonymous mutations (also termed recoding). Case studies have revealed the essential roles of nonsynonymous RNA editing in multiple biological aspects such as environmental adaptation of *Drosophila* [21], temperature tolerance of octopuses [22], and developmental regulation of mice [23, 24]. Therefore, these case studies leave an impression that RNA editing events, particularly the nonsynonymous sites, are positively selected as they diversify the proteome in a temporal-spatial manner, circumventing the pleiotropic effect of DNA mutations.

Apart from these case reports on functional recoding sites, a more basic question for evolutionary biologists is whether we can observe signals of adaptation/positive selection for the genome-wide nonsynonymous editing sites. Among the various metazoans, those species with overrepresented nonsynonymous editing are distributed in two major clades, the coleoids of cephalopods (octopus, squid, and cuttlefish) [14, 25] and insects like *Drosophila* and honeybees [26, 27]. It is worth thinking when did the extensive recoding sites emerge and how did they evolve. For cephalopods, it is already known that the early-diverging nautilus and sea hare bear few recoding sites and therefore the prevalent recoding was an invention in coleoids [14]. For insects, however, the species with systematic RNA editing studies only covered a small corner compared with the large set of insect species.

### RNA editing in insects and the importance of studying Heteroptera (Hemiptera)

Insects are the most diversified clade in the animal kingdom. The ancestor of insects experienced gene loss and the extant insects encode a single *Adar* gene [19] which is homologous to the mammalian *ADAR2* gene [28]. *ADAR*s mainly expresses in neuronal tissues and therefore A-to-I RNA editing is most prevalent in neuronal genes [14, 25, 29–31]. Among the three ADAR proteins in mammals (ADAR1, ADAR2, and ADAR3), ADAR2 would preferentially edit the mRNA (genic) region [32]. Therefore, the Adar homolog in insects is also expected to mainly target the mRNA region. Accordingly, an excessively high fraction of editing sites in coding genes was found in *Drosophila* compared to the scarcity of mammalian editing sites located in genic regions [33, 34].

**Fig. 1:**
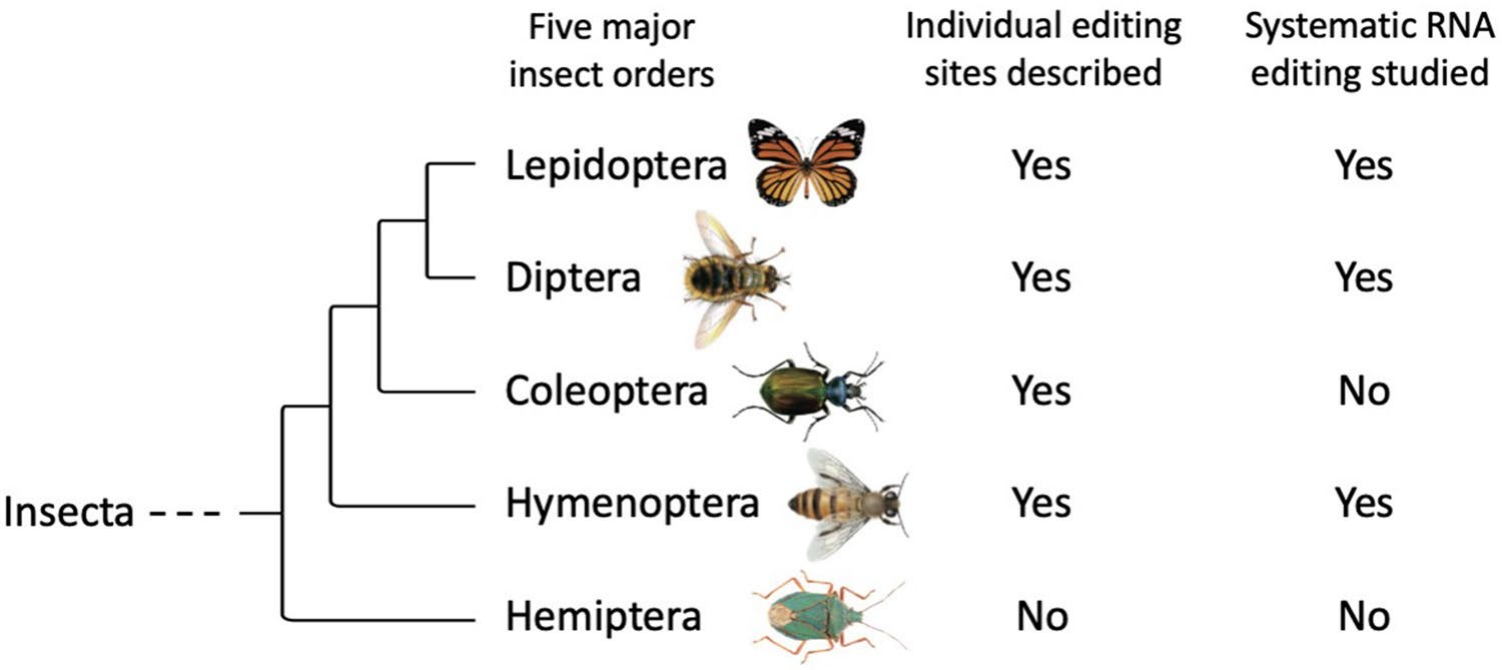
An overall record of A-to-I RNA editing studies in five major insect clades. Hemiptera is the earliest-diverging clade of the five orders, while no editing studies were performed in Hemiptera

Coleoptera, Lepidoptera, Hymenoptera, Diptera, and Hemiptera are the five insect orders with the greatest numbers of species (Fig. 1). To date, the insects of the top four orders have been studied on A-to-I RNA editing either by systematic transcriptomic analyses [33, 35–38] or cases studies of individual RNA editing events [39–43]. Particularly, for Diptera and Hymenoptera, the RNA editomes of multiple species have been systematically investigated [27, 33, 34, 36, 37], enabling researchers to find conserved and non-conserved editing sites and infer their evolutionary significance. For example, nonsynonymous RNA editing is overrepresented and highly conserved across *Drosophila* species, suggesting the potential benefits conferred by recoding [27, 33, 44]. Moreover, long-distance convergent evolution of recoding sites between *Drosophila* and bees indicated the need for recoding the neuronal genes in insect brains [26].

Hemiptera is the earliest-diverging clade of the five major insect orders (Fig. 1). The suborder Heteroptera (true bugs) represents the most successful incomplete metamorphosis insects [45]. Heteroptera species have amazingly high phenotypic/behavioral diversities at both inter-species level (variety) and intra-species level (plasticity). They have adapted to a wide variety of habitats and evolved different feeding traits [46–48]. However, the genetic and molecular mechanisms governing this phenotypic diversity remains unknown. The key questions are, is this diversity/plasticity achieved at genomic or epigenomic level? Could this molecular diversity be formed beyond the DNA sequence? How prevalent is A-to-I RNA editing in Hemiptera species? How RNA editing affects the transcriptomic plasticity under different environmental conditions? The early-diverging Hemiptera serves as a valuable resource that helps infer the landscape of RNA editomes in unexplored insects. Given the overall prevalent nonsynonymous editing in *Drosophila* (Diptera) and honeybees (Hymenoptera), it remains unclear whether the extensive recoding exists in the ancestor of all insects or it was independently gained in lately-diverging clades. Thus, there is adequate motivation to study the A-to-I RNA editing in Hemiptera species.

### Amis and scopes

In this work, we aim to investigate the following key questions: (1) What is the RNA editing landscape in representative Hemiptera species? (2) Does overrepresented recoding exist in this early-diverging insect order? (3) Upon environmental changes or stress, does RNA editing contribute to the phenotypic and molecular diversity/plasticity in Hemiptera insects? If so, how does RNA editing regulate the diversity and plasticity?

*The jiuxiang bug Coridius chinensis* (Hemiptera: Heteroptera: Dinidoridae) is widely used as traditional Chinese medicine to treat various kinds of pains, nephropathy, male dysfunction, stomach cold, and many other diseases [49–53]. Physiologically, *C. chinensis* is able to tolerate relatively low temperature and can autonomously transfer to the diapause status in winter. It is intuitive to ask how the transcriptome and proteome of *C. chinensis* are regulated to achieve the plasticity? Together with the RNA editing-related questions raised above, there is urgent need in understanding the mechanism of molecular complexity in *C. chinensis* beyond the genome sequence. We previously sequenced the mitochondrial genome and transcriptome of *C. chinensis* and found a distinct mode of mitochondrial transcription [54]. Here, we assembled the complete chromosome-level reference genome of *C. chinensis*, and further sequenced the head transcriptomes and the matched DNA resequencing of ten *C. chinensis* samples, with five under room temperature (26°C) and five under cold stress (10°C). We depicted the gene expression profiles and RNA editomes of each sample. Like our previous findings in *Drosophila* and bees [26, 55], we again found that in *C. chinensis* the nonsynonymous RNA editing events were overrepresented compared to synonymous ones, suggesting that extensive recoding exists in early-diverging insect order(s). Prevalent intronic editing was also identified. However, only very few recoding sites in well-known neuronal genes were conserved across multiple orders, indicating the independent gain of species-specific editing sites during evolution. Under cold stress, the global editing efficiency was unexpectedly down-regulated, potentially explained by the “supply matches demand” theory upon the shut-down of general transcriptional processes. Nevertheless, we found an interesting site with “conserved editing but non-conserved recoding” in potassium channel *Shab* which was significantly up-regulated in cold, serving as a candidate functional site in response to temperature stress. In conclusion, the first RNA editome in Hemiptera has greatly advanced our understanding on the evolution and adaptation of A-to-I RNA editing.

## Materials and methods

### Sample collection and sequencing for constructing reference genome

*Coridius chinensis* was collected from Leshan, Sichuan, China (N29.52°, E103.43°). A single female adult of *C. chinensis* was prepared for de novo sequencing. Genomic DNA was extracted using the CTAB method, followed by purification using a Blood and Cell Culture DNA Midi Kit (QIAGEN, Germany). The genome assembly was performed using a hybrid sequencing approach, combining SMRT PacBio High-Fidelity (HiFi) reads, Illumina short reads, and Hi-C data. A long fragment library with an average insert size of approximately 15 kb was constructed from the extracted DNA. HiFi reads were generated using a PacBio Sequel sequencer (Pacific Biosciences, Menlo Park, USA), and Hi-C data were generated by Illumina NovoSeq platform. Additionally, RNA-Seq reads were generated from one male and one female using Illumina Novoseq platform. All library construction and sequencing procedures were performed at Grandomics Biotechnology Co., Ltd (Wuhan, China).

For Hi-C sequencing, fresh tissues were obtained from a female individual of *C. chinensis*. The sample was cross-linked with formaldehyde isolation buffer, and then digested with DpnII restriction endonuclease. After ligation, the DNA fragments were split into a size of 350-bp, and the chromatin conformation capture library was sequenced on an Illumina NovoSeq platform.

### Sample collection for head transcriptome and matched genome resequencing

*Coridius chinensis* samples were collected in Ankang, Shaanxi Province, China (108.32°E, 33.32°N). All samples were housed in controlled environments with 30 cm × 40 cm × 50 cm cages situated in the laboratory. The insects were reared on fresh pumpkin seedlings to ensure their growth and development. The *C. chinensis* samples were divided into two groups: room temperature (control, 26°C) and low temperature (cold stress, 10°C). Each group comprised five samples: two adult females, two adult males, and a mixed sample of one adult female and one adult male. Control group was maintained at a constant environmental of 26°C, while cold stressed group was placed at 10°C for 24 h. All samples were kept with a relative humidity of 70 ± 5%. Following a 24-h treatment period, the insects were rapidly frozen in liquid nitrogen for subsequent procedures. For the insects used in the last section of Results, all conditions are the same except that we treated them in 10°C for 30 days. For this batch of insects, we only extracted their head RNA/DNA for Sanger validation and no RNA-Seq library was constructed.

Head of each individual (sample) was used to construct an RNA-Seq library, and the matched body of each sample was subjected to DNA-resequencing. For the mixed female and male sample, the heads of the two individuals were pooled for RNA-Seq and the matched bodies of them were pooled for DNA-Seq. Total RNA extraction was performed using TRNzol Reagent Kit. Genomic DNA extraction utilized the CTAB method, followed by purification using a Blood and Cell Culture DNA Midi Kit (QIAGEN, Germany). Subsequently, RNA-Seq and DNA-Seq libraries were constructed and sequenced on the Illunina NovoSeq 6000 platform at Berry Genomics Biotechnology Co., Ltd. (Beijing, China).

### Genome assembly

We assembled a primary contig genome in wtdbg2 v2.5 using default parameters [56]. Then, we used the Purge_dups v1.2.3 [57] tool to remove heterozygous duplication and improve continuity. Next, we applied a scaffolding pipeline based on Durand (2016) to generate a high-quality chromosome-scale genome [58]. In brief, we mapped Hi-C data to the contig assembly in BWA-MEM v0.7.17 [59], created DpnII sites in Juicer v1.5 [58], and built primary scaffolds by the 3D-DNA v180922 [60]. We visualized and manually curated the assembly using Juicebox Assembly Tools v1.9.8 [61] before processing another round of scaffolding using 3D-DNA v180922 [60]. Then, the final chromosome genome assembly was obtained.

We evaluated the completeness of the assembled genome using Benchmarking Universal Single-Copy Orthologs (BUSCO v3.0.2) at the insect (insecta_odb10) level [62]. Additionally, we assessed the assembly accuracy by mapping short reads to the genome in BWA-MEM v0.7.17 [59] and estimating the base error using quality value scores in Merqury v1.1 [63].

### Genome annotation

Repetitive elements in *C. chinensis* genome were identified using RepeatMasker v4.0.7 [64] and RepeatModeler v2.0.1 [65]. Long terminal repeat retrotransposons (LTR-RTs) were detected using LTRFinder v1.06 [66]. Tandem repeats were annotated by Tandem repeats finder v4.07b [67].

Genes in the assembled genome were predicted using a combination of homology-based, transcriptome-based, and de novo strategies. Homology-based predictions involved downloaded homologous proteins and transcripts from several species, including *Apolygus lucorum*, *Cimex lectularius*, *Orius laevigatus*, *Rhodnius prolixus*, *Triatoma rubrofasciata,* and *Drosophila melanogaster* (NCBI, https://www.ncbi.nlm.nih.gov/; InsectBase v2.0) [68]. The homologous proteins and transcripts were then aligned in Exonerate v2.4.0 for training gene sets [69]. Additionally, a sorted and mapped bam file of RNA-seq data was converted to a hint file using the bam2hints program in AUGUSTUS v3.2.3 [70]. The self-trained sets were combined with hint files as inputs for AUGUSTUS v3.2.3 to predict de novo coding genes from the assembled genome [70]. Finally, the homology-based, de novo-derived, and transcript genes were merged in MAKER v2.31.10 to generate a high-confidence gene set [71].

Gene structure and annotations were determined using eggnog-mapper v2.0.1 [72], InterProscan v5.0 [73], BLAST v2.2.28 [74], and HMMER v3.3.2 [75] to search against Non-Redundant Protein Sequence Database (NR), Gene Ontology (GO), Clusters of Orthologous Groups of Proteins (COG), Kyoto Encyclopedia of Genes and Genomes (KEGG), Swiss-Prot, and Pfam databases.

### Mapping and variant calling

RNA-Seq reads were mapped to the *C. chinensis* reference genome using STAR v2.4.2 with default parameters [76]. DNA-Seq reads were mapped to the genome using BWA-MEM v0.7.17 [59]. Uniquely mapped reads were maintained and then duplicated reads (from PCR) were removed. Variants were called with GATK [77] haplotype caller by requiring base quality Q > 30. The bases located in 10 bp of reads ends were removed (after alignment). Soft-clipping bases were removed from the reads.

Soft-clipping refers to the situation where only part of the reads is aligned to the reference sequence, and the unmapped part was termed soft-clipped, labeled as “S”. The BAM file (the reads alignment file) contains a column of CIGAR. For example, for a 150 bp read, CIGAR = 150 M means that all 150 bps were completely mapped to the reference. 120M30S means that the first 120 bps were mapped and the last 30 bps were unmapped (for any reasons). 30M120S means that the first 30 bps were mapped and the last 120 bps were unmapped. Thus, removing the soft-clipped bases means removing all the bases labeled as “S” in the alignment, no matter how long the S is. In contrast, removing 10 bp at both ends essentially considers that sequencing errors are likely occur at both ends, regardless of whether the 10 bps were labeled as M or S. In brief, removal of 10 bp considers the sequencing quality while removal of soft-clipping considers the alignment issue.

**Fig. 2:**
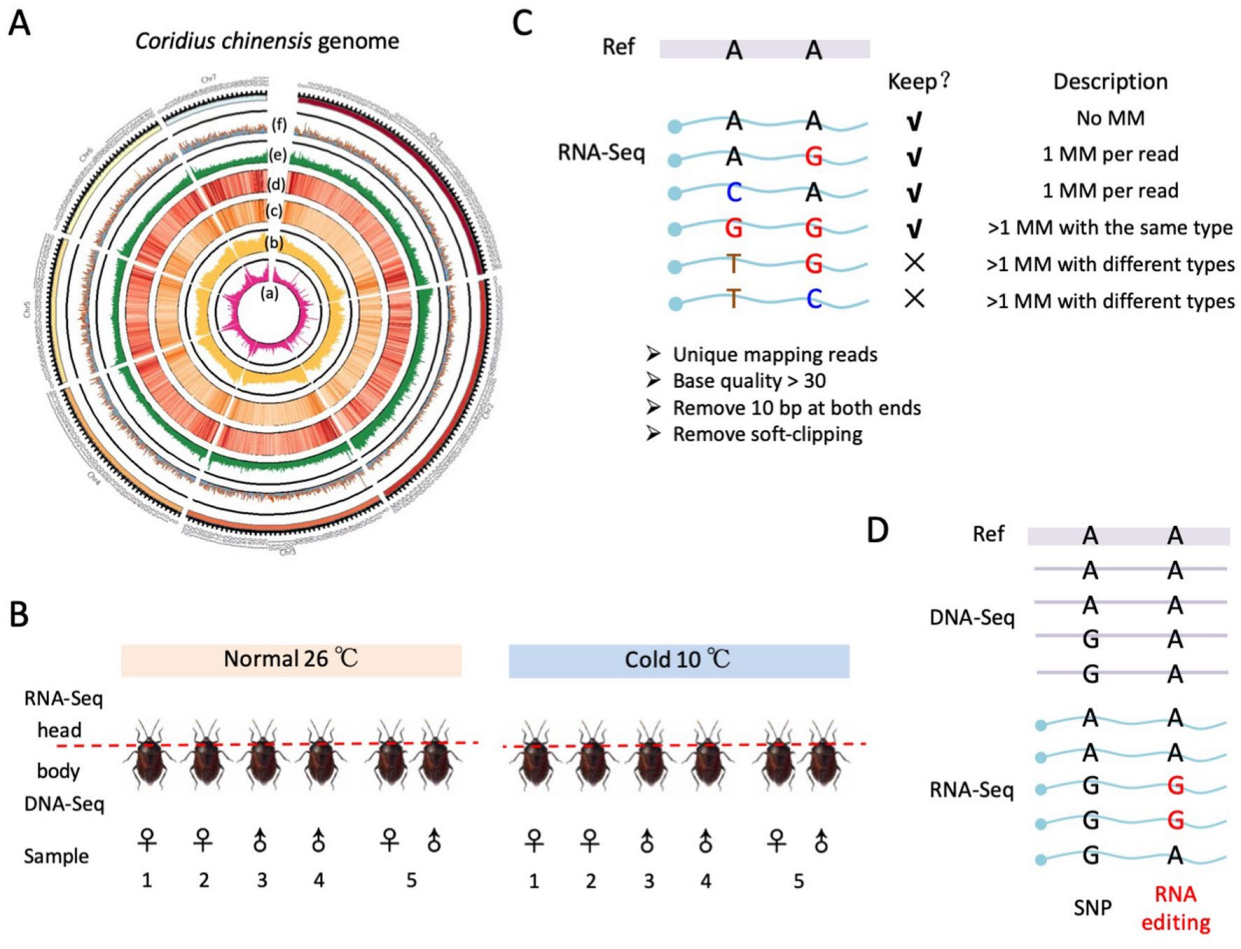
Genome assembly and identification of A-to-I RNA editing sites in *Coridius chinensis*. **A** Circos plot of the genome. a, tandem repeats; b, all transposable elements; c, long terminal repeats; d, DNA transposon; e, GC content; f, coding genes. **B** Schematic diagram illustrating the overall design and the samples used in this study. **C** A diagram introducing how we treat the mismatches in RNA-Seq data. **D** Distinguishing between RNA editing sites and SNPs using both DNA-Seq and RNA-Seq

Then, we only kept the reads with no more than one mismatch, or with more than one mismatch of the same type. For example, reads with one A > G variant and one A > T variant were discarded, but reads with two or more A > G variants were allowed (Fig. 2). On each genomic site, the numbers of reads supporting the reference allele (ref) and alternative allele (alt) were recorded for the downstream analysis.

### Identification of RNA editing sites

In our previous works [26], we defined a binomial test to calculate the probability of obtaining a variant site by sequencing error, termed *P*_*error*_. This parameter was based on the sequencing coverage on a particular site together with the reads supporting the alternative allele. For each variant site in each library, the *P*_*error*_ was adjusted by multiple testing correction [78] to obtain an FDR. The default parameter in R package p.adjust(x, method = “fdr”) was used, where the default N = length(x) means the number of total variations in RNA-Seq in our case. Each sample had both RNA-Seq and matched DNA-Seq, a reliable RNA editing site (also termed RNA–DNA difference, RDD) in a sample would meet the following criteria: (1) FDR < 0.05 for the variants in RNA-Seq; (2) DNA-Seq has coverage ≥ 10 and no alternative alleles were detected in DNA-Seq. The RDDs appeared in any of the ten samples were regarded as candidate RNA editing sites. Since the RDDs were reliable given the availability of RNA-Seq and matched DNA-Seq, we did not require an editing site to appear in at least two or more samples, that over-stringent criterion would exclude potential true positive sites. As we have shown in the results, 2904 (72.4%) of the RDDs were A-to-G, representing reliable A-to-I RNA editing sites.

Note that since our RNA-Seq libraries were non-strand-specific, the reads from intergenic regions cannot be assigned to a particular strand. That means, if the annotated intergenic region is indeed transcribed (for unknown reasons), then we cannot figure out whether these RNAs come from positive strand or negative strand, let alone understand the potential variations on these RNAs. Thus, we only focused on the variations in annotated genes. Among the 4009 RDDs in genic regions, 2904 (72.4%) of them are A > G variations, suggesting the reliability of the editing detection and filtering pipeline.

Notably, we did not distinguish regular editing and hyper-editing for the following reasons. (1) The two terminologies do not have clear boundaries because hyper-editing sites are typically detected from the reads unmapped by regular aligners like BWA [79]. The more mismatches allowed, the more reads will be mapped by regular aligners. (2) We used STAR that allows as many as 15% × N mismatches (N = read length), making a 150 bp read mappable with even 22 mismatches [76, 80]. This largely enables the detection of the so-called hyper-editing reads. In the section describing highly clustered intronic sites, we have manually inspected many hyper-edited reads in intronic regions. Sanger sequencing also validated the clustered editing sites. Thus, our results should be reliable and they should include both regular editing and hyper-editing. We acknowledge that the unmapped and transform strategy [79] might retrieve additional hyper-editing sites, but those sites were usually of low coverage and were unlikely to be identified as differential editing sites (see below). We did not pursue a huge number of editing sites, and instead we tried to identify editing sites with sufficient coverage together with those differential editing sites. As we will show in the results, the conserved recoding sites in neuronal genes (with high coverage and are verified by IGV and Sanger sequencing) are highly detectable with regular procedures, and are unlikely to be missed due to the lack of hyper-editing pipeline.

To confirm the robustness of A > G% and the rationale of using these cutoffs, we also tried other cutoffs of FDR, N, number of samples showing editing, and DNA coverage. We tried several FDR values higher or lower than 0.05 (Supplementary Fig. S1A), N values of genic region size (Supplementary Fig. S1B) or genome size (Supplementary Fig. S1C), and several DNA coverages lower or higher than 10 (Supplementary Fig. S1D). The results showed that different cutoffs produced similar A > G%, with larger N value producing slightly higher A > G% and lower number of total sites. Since our default cutoffs produced an acceptable signal-to-noise ratio compared to stricter or looser cutoffs, we used these traditional cutoffs. Notably, by requiring at least two samples having editing, the A > G% even slightly decreased (68.9%) and the number of total A > G sites (1189) was largely reduced. This suggests that the requirement for editing events being detected in multiple samples does not increase accuracy but might miss many true positive sites.

### Differential editing sites (DES)

We defined DES between normal (26°C) and cold (10°C) samples using a combined method with Fisher’s exact test and five versus five T-test. First, reads from the five normal or five cold samples were pooled, respectively. For each editing site, the numbers of reads supporting the reference A allele (ref) and alternative G allele (alt) were recorded, denoted as ref_N_, alt_N_, ref_C_, and alt_C_, where subscript N stands for “normal” and C stands for “cold”. Fisher’s exact test was exerted to the four numbers (ref_N_, alt_N_, ref_C_, and alt_C_) to calculate a *P* value followed by multiple testing correction [78]. Sites with FDR < 0.05 were maintained. However, uneven coverages of different libraries might introduce biases to highly covered libraries, which is, DES is likely to appear at the sites in highly covered samples/regions. To fix this bias, the editing levels in each single sample should be considered. Therefore, we further performed T-tests on editing levels of five normal *versus* five cold samples and required FDR < 0.05 with the same direction of the pooled level comparison. Sites passed the two steps were regarded as DES.

### Linear regression analysis

With the RNA editomes of 10 samples with 24 h treatment, we performed linear regression analysis on editing level against temperature (variable 1) and gender (variable 2). The R package lm was used. The code is summary (lm(Y ~ X1 + X2)), where Y is editing level in each sample, X1 is temperature (0 denotes normal and 1 denotes cold), and X2 is gender (0 denotes male and 1 denotes female). The output *P* value of each variable will indicate whether this variable significantly contribute to Y. The linear regression was performed with all ten samples, or without the two mixed female and male samples.

### Annotation of RNA editing sites and the expected Nonsyn/Syn ratio

The RNA editing sites were annotated with SnpEff [81], which tells us whether a variation site is located in intergenic region, genic region, intron, UTR, CDS, causing a nonsynonymous (Nonsyn) or synonymous (Syn) mutation. The expected Nonsyn/Syn ratio was calculated by changing every adenosine into guanosine in the *C. chinensis* genome, but the Adar motif was considered. First, the 3-mer motif of the ~ 3000 A-to-I RNA editing sites were extracted, and we counted the 16 combinations of the –1 and + 1 nucleotide, recording their proportions. Next, for all the unedited adenosines in the CDS (totally 7,389,019 unedited adenosines), we sampled equal proportions of the adenosines with each of the 16 combinations but letting their total amount be 7,389,019. Then, we calculated the Nonsyn/Syn ratio of these 7,389,019 sampled adenosines to be the expected Nonsyn/Syn ratio of A-to-I RNA editing.

### RNA structure prediction

The hairpin structures in the pre-mRNAs were identified using RNALfold [82] with default parameters. Different cutoffs of minimum free energy (MFE) were tried. Regions meeting “MFE < cutoff” were defined as hairpin structures. The folding and visualization of RNA structures of a given sequence was accomplished by the “RNAstructure” website (https://rna.urmc.rochester.edu/RNAstructureWeb/).

### Differential expression analysis

Reads count for each gene in each sample was accomplished by featureCounts [83]. Differential expression was done by DESeq2 with default settings [84]. The comparison between five normal versus five cold samples was carried out. Genes with FDR < 0.05 were regarded as differentially expressed genes (DEG), making up ~ 7.8% of the total genes. Since DEG was not our main focus, we did not try different cutoffs on log_2_foldchange to define DEG. Instead, for particular gene of interest (e.g. *Adar*), we directly checked the *P* value, FDR, and log_2_foldchange value to judge how confident it was to be a DEG.

### Random shuffling and randomization test

Let N0 = number of edited genes with at least one intron (with > 1 exons).

N1 = number of genes with recoding sites among the N0 genes.

N2 = number of genes with intronic editing.

We randomly sampled N1 genes from those N0 genes, and simultaneously sampled N2 genes from those N0 genes. Sampling was done with replacement. The overlap between the sampled N1 and N2 genes were recorded. This procedure was repeated for 1000 times to get 1000 numbers. Only 3 out of the 1000 numbers > 9, where 9 is the observed overlap between the recoding genes and intron edited genes. Then, the *P* value for randomization test was 0.003.

### Annotation, folding, and visualization of protein domains and structures

We constructed the CDS sequences with intron retention and translated them into protein sequences. The protein domains and families of the inserted and un-inserted versions were identified using the NCBI Conserved Domain Database (CDD) (www.ncbi.nlm.nih.gov/-Structure/cdd/cdd/shtml). The resulting diagrams of protein domains were visualized using TBtools v1.108 [85], a biosequence structure illustrator. The protein secondary structure was visualized using PSIPRED program [86]. AlphaFold was performed by running the AlphaFold2 notebook on Google Collaboratory cloud computing facilities with default parameters. The Google Colab is accessible online at https://colab.research.google.com/github/phenix-project/Colabs/blob/main/alphafold2/AlphaFold2.ipynb. The resulting models were displayed with the PyMOL molecular graphics system [87].

### Sanger sequencing validation

To validate whether the candidate sites are edited and confirm the editing level, we employed Sanger sequencing on PCR-amplified genomic DNA (gDNA) and cDNA sequences. For cDNA synthesis, 500 ng of total RNA was revered transcribed using PrimeScript™ RT reagent Kit with gDNA Eraser Kit (TaKaRa), following the manufacturer’s instructions. Primer sequences are listed in Supplementary Table S1. Typically, a 25 µl PCR reaction comprised EmeraldAmp® Max PCR Master Mix (TaKaRa), 100 ng of gDNA (or 5 ng of cDNA) template, and 10 μM each of forward and reverse primers. The PCR program was set as: 95°C for 1 min, followed by 40 cycles of 95°C for 20 s, 54°C for 30 s, and 68°C for 30 s, with a final extension at 72°C for 5 min. Primers were synthesized in Sangon Biotech (Shanghai) Co., Ltd., and Sanger sequencing was conducted by Beijing Tsingke Biotech Co., Ltd. Evaluation of RNA editing involved measuring peaks heights from Sanger sequencing traces using SnapGene software (https://www.snapgene.com/).

## Data availability statement

All data generated by this study was uploaded to NCBI. For genome sequencing data, the accessions numbers are SRR23604985 (RNA-Seq), SRR2360984 (Illumina short read), SRR23604986 (PacBio-HiFi read), and SRR23604983 (Hi-C data). The assembly genome was accessible in GenBank with accession ID JARDVX000000000. The RNA-Seq and the DNA-Seq data for both wild control and low temperature samples were available under accession number SRP476000. The Sanger sequencing data were included in Supplementary Data 1 (24 h treatment) and Supplementary Data 2 (30 d treatment).

## Results

### Genome assembly of Coridius chinensis

We used 51.5 Gb of highly accurate long-read (HiFi) reads, 57.2 Gb of short reads, and 129.6 Gb of Hi-C data generated in this study to assemble the *C. chinensis* genome (Supplementary Table S2). The assembled genome had a size of 1.40 Gb with seven complete chromosomes (Fig. 2A, Methods), an N50 of 209.1 Mb, an overall GC content of 33.6%, a completeness of 94.4%, a quality value of 32.7, and a short-read alignment rate of 99.3% (Supplementary Table S3, see Methods for the detailed description). These parameters suggested that the quality of the genome is sufficiently high to perform the downstream analyses. Next, our genome annotation revealed 24,728 protein-coding genes (Supplementary Table S4) and most of these genes (94.5%) were successfully annotated using at least one public database (Supplementary Table S5). Then, different types of transposable elements were also identified in the genome (Supplementary Fig. S2).

### The C. chinensis genome encodes a single Adar gene

The annotated *C. chinensis* genome contains a single *Adar* gene homologous to *Drosophila Adar* (*dAdar*) and mammalian *ADAR2*. The *C. chinensis* Adar protein has a length of 591 AAs (Supplementary Fig. S3), with two dsRNA-binding domains located at N-terminal (AA positions 21–79 and 135–180) and one deaminase domain located at C-terminal (AA positions 276–578). Comparably, the canonical *D. melanogaster* Adar protein is 667 AAs long and the domains are located at AA positions 56–118, 201–247, and 294–665. This suggests the high conservation level of Adar sequence, length, and domain architecture in insects.

### Identification of RNA editing sites in heads of C. chinensis

We treated the bugs under normal (26°C) or cold stress (10°C) for 24 h. Under each temperature, we generated five samples including two female individuals, two male individuals, and a mixed sample of one female + one male. Head of each individual was used to construct an RNA-Seq library, and the matched body of each was subjected to DNA-resequencing (Fig. 2B). For the mixed female and male sample, the heads of the two individuals were pooled for RNA-Seq and the matched bodies of them were pooled for DNA-Seq (Fig. 2B). On average, we obtained 7.45 Gb RNA-Seq and 22.72 Gb DNA-Seq data for each of the ten libraries, covering an average genome-wide depth of 33.12 × and 16.25 × , respectively (Table 1).

**Table 1:** Sequencing depth of *C. chinensis* samples generated by this study

Individual	Data size (Gb)		Genome-wide coverage ( ×)	
	RNA-Seq (head)	DNA-Seq (body)	RNA-Seq (head)	DNA-Seq (body)
Normal 1 F	3.62	20.59	16.08	14.73
Normal 2 F	4.33	20.25	19.23	14.48
Normal 3 M	3.81	22.05	16.95	15.77
Normal 4 M	4.04	21.58	17.98	15.43
Normal 5 F + M	3.95	21.58	17.56	15.43
Cold 1 F	3.55	22.02	15.80	15.75
Cold 2 F	4.12	26.45	18.34	18.92
Cold 3 M	4.19	21.51	18.62	15.38
Cold 4 M	3.74	27.32	16.62	19.54
Cold 5 F + M	4.36	23.87	19.39	17.07

RNA-Seq was generated by individual heads and DNA-Seq was generated by the body of matched individual

The data size of RNA-Seq was the number of reads after duplications were removed

The RNA-Seq coverage was calculated by data size divided by the total length of genic region

*F* female, *M* male

We used stringent criteria to filter the reads and mismatches in the RNA-Seq data to ensure that those mismatches seen in RNA-Seq were not sequencing errors or artifacts from misalignments (Fig. 2C and Materials and Methods). For a particular site in a given sample, if the variants in RNA-Seq passed the binomial test (see Materials and Methods) and meanwhile all the DNA reads (≥ 10) supported the reference allele, then we regarded this site as a candidate RNA–DNA difference (RDD) in this sample (Fig. 2D). The sites with bi-allelic DNA coverage or without DNA-Seq covered were not considered. Then, the final set of RDD, representing reliable RNA editing sites, was defined as the candidate RDD sites appeared in any of the ten samples (Materials and Methods).

**Fig. 3:**
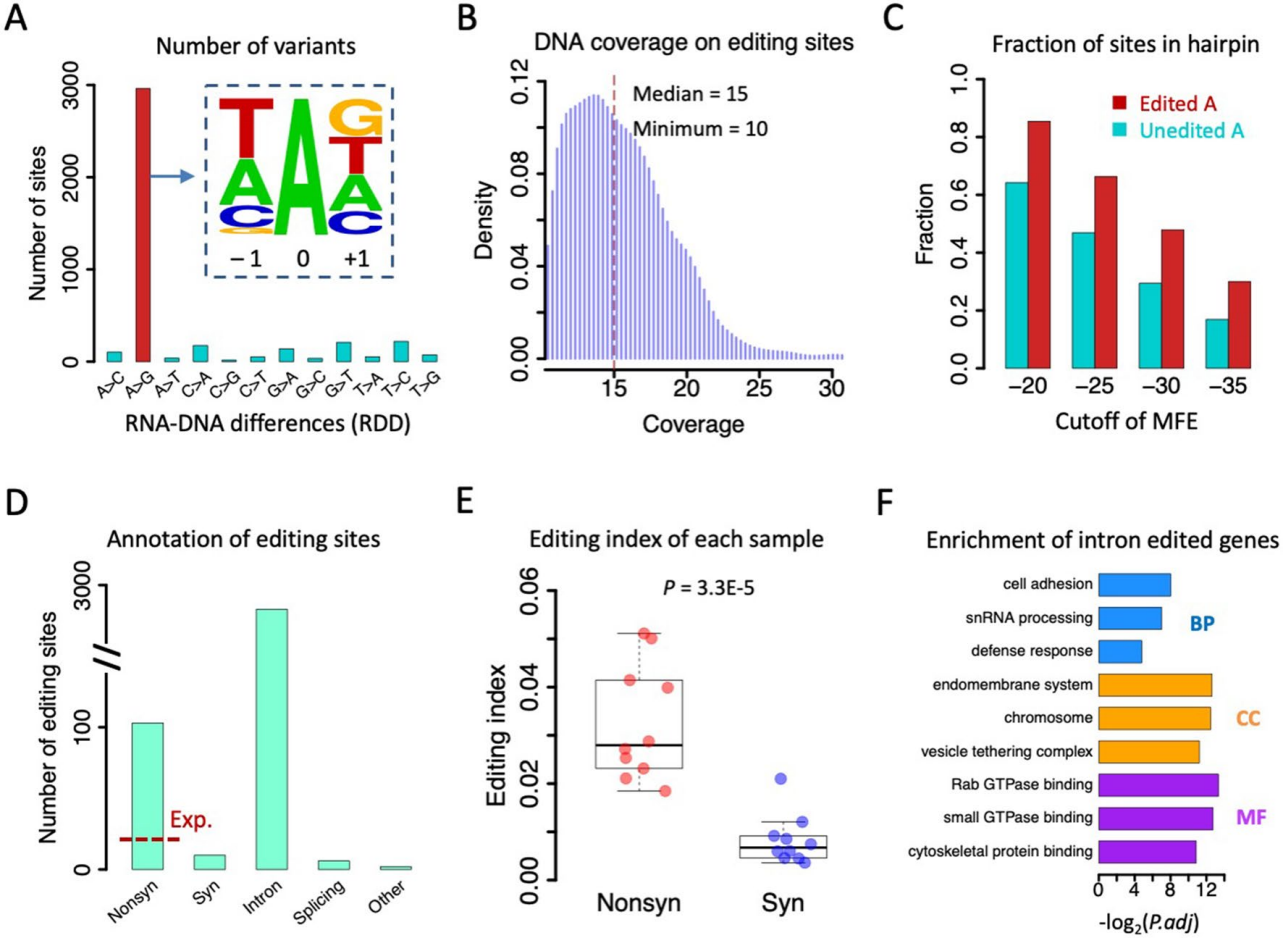
A-to-I RNA editome of *Coridius chinensis*. **A** Barplots showing the numbers of 12 types of RDD in genic regions. The 3-mer motif of the A > G variation sites was shown in the small panel. **B** Density plot of DNA coverage on A-to-I RNA editing sites in each sample. **C** Fraction of sites located in hairpin structures. Edited and unedited adenosines were calculated separately. Different cutoffs of MFE were used to define the hairpin regions. **D** Annotation of genic A-to-I RNA editing sites. The red dashed line represents the expected number of nonsynonymous editing sites. **E** Boxplot displaying the editing index of nonsynonymous and synonymous sites in ten samples. Sites with ≥ 20 RNA coverage were used. *P* value was calculated by paired *T*-test. **F** Gene ontology of genes with intronic RNA editing sites. *BP* biological process, *CC* cellular component, *MF* molecular function

Since our RNA-Seq libraries were non-strand-specific, the reads from intergenic regions cannot be assigned to a particular strand and thus we only focused on the variations in annotated genes. We totally identified 4009 RDDs in genic regions and found 2904 (72.4%) of them are A > G variations (Fig. 3A). This A > G fraction was 13.3 times more abundant than the second highest variant type T > C (Fig. 3A), suggesting the high confidence of regarding these 2904 A > G variants as A-to-I RNA editing sites (Supplementary Table S6). We also checked the A > G% in different genomic regions and saw that A > G% was ~ 72% for both intronic sites and nonsynonymous sites (Supplementary Fig. S4), but was higher in genomic repeats (2144 A > G sites, 85.1% of total variations, 13.1 times higher than the 2nd highest variation) compared to non-repeats (760 A > G sites, 51.0% of total variations, 4.6 times higher than the 2nd highest variation) (Supplementary Fig. S4), presumably due to the hyper-editing events in repeats. The slight fluctuation of A > G% in different genomic regions does not affect the overall reliability of these 2904 A-to-G(I) sites as we observed the 3-mer motif around these sites well agreed with the known ADAR motif in animals (Fig. 3A), where the upstream nucleotide avoids G and the downstream nucleotide prefers G. Since we required DNA coverage ≥ 10 in each sample, all editing sites were supported by at least 10 reads without alternative allele in DNA-Seq, and the median DNA coverage was 15 per sample (Fig. 3B). The sufficient DNA coverage excluded the potential SNPs that confounded the RNA editing profile, increasing the authenticity of identified RNA editing sites. To further show the reliability of RNA editing, we calculated the fraction of sites located in predicted hairpin structures in the pre-mRNA, finding that the 2904 A-to-I RNA editing sites were constantly enriched in dsRNA structures compared to unedited adenosines (Fig. 3C), agreeing with the known ADAR preference.

### Signals of adaptation in RNA editome of C. chinensis

Next, we annotated the 2904 A-to-I editing sites in genic regions. We obtained 103 nonsynonymous sites, ten synonymous sites, 2783 intronic sites, six sites in splicing region, and two sites in UTRs (Fig. 3D). The fraction of sites located in repeats were 75.5% for intronic sites, 35.0% for nonsynonymous sites, and 50.0% for the few synonymous sites (Supplementary Fig. S5). Among the total 2904 editing sites, we first noticed that the Nonsyn/Syn ratio was 103/10 = 10.3, and if we randomly sampled the unedited adenosines considering the editing motif, the Nonsyn/Syn was 1.79 for A-to-G mutations (Materials and Methods). The observed nonsynonymous editing was 5.8 times higher than expectation (Fig. 3D), which was a strong indication that the A-to-I recoding sites were beneficial and positively selected. If we only focus on non-repetitive regions, the observed-to-expected ratio of Nonsyn/Syn editing will be even more impressive (observed Nonsyn/Syn = 67/5 = 13.4; expected Nonsyn/Syn = 1.77; foldchange = 13.4/1.77 = 7.6 times). Moreover, we calculated the editing index = ΣG/(ΣG + ΣA) of the sites with RNA-Seq coverage ≥ 20 in each sample, and found that nonsynonymous sites had significantly higher editing efficiency than synonymous sites (Fig. 3E). These signals of beneficial recoding were also observed in *Drosophila* and honeybees [26, 27], suggesting that (1) Overrepresentation of A-to-I recoding might be prevalent in different insect clades; and (2) The signal of beneficial recoding exists in this early-diverging insect order Hemiptera.

We then performed gene ontology (GO) enrichment of the genes bearing RNA editing sites. Since most editing sites are located in introns, we will first look at these intron-edited genes and then describe the CDS-edited genes with particular examples. 831 genes had intronic editing and on average each of them had 3.4 intronic editing sites. In contrast, 102 genes had CDS editing sites and on average each of them had 1.1 editing sites. Virtually only six genes had more than one editing site in CDS. These results conform to the notion that coding editing sites were less likely to appear in clusters compared with non-coding editing sites. Interestingly, intron editing was not enriched in neuronal genes but showed significant preference in genes related to defense response, GTPase and cytoskeleton binding (Fig. 3F). This raises a possible role of RNA editing in metabolism and dynamic regulation in environmental adaptation or stress response of insects.

### Neuronal genes with conserved and species-specific recoding sites

We set out to investigate the genes with CDS editing especially nonsynonymous editing sites. Since the recoding sites were overall beneficial, we wondered whether we could find long-distance conservation of recoding sites or recoded genes between *C. chinensis* and *Drosophila*. We totally found five genes with recoding events in both species (but the editing sites were not necessarily conserved): *Shab* (*Shaker cognate b*), *Sh* (*Shaker*), *Ank2* (*Ankyrin 2*), *capu* (*cappuccino*), and *Unc-89* (*Obscurin*). Three (*Shab*, *Sh*, and *Ank2*) out of five genes were nervous system-related. Two genes *Shab* and *Sh* possessed recoding sites with editing level > 0.5, while the editing levels in the other three genes were lower than 0.1.

**Fig. 4:**
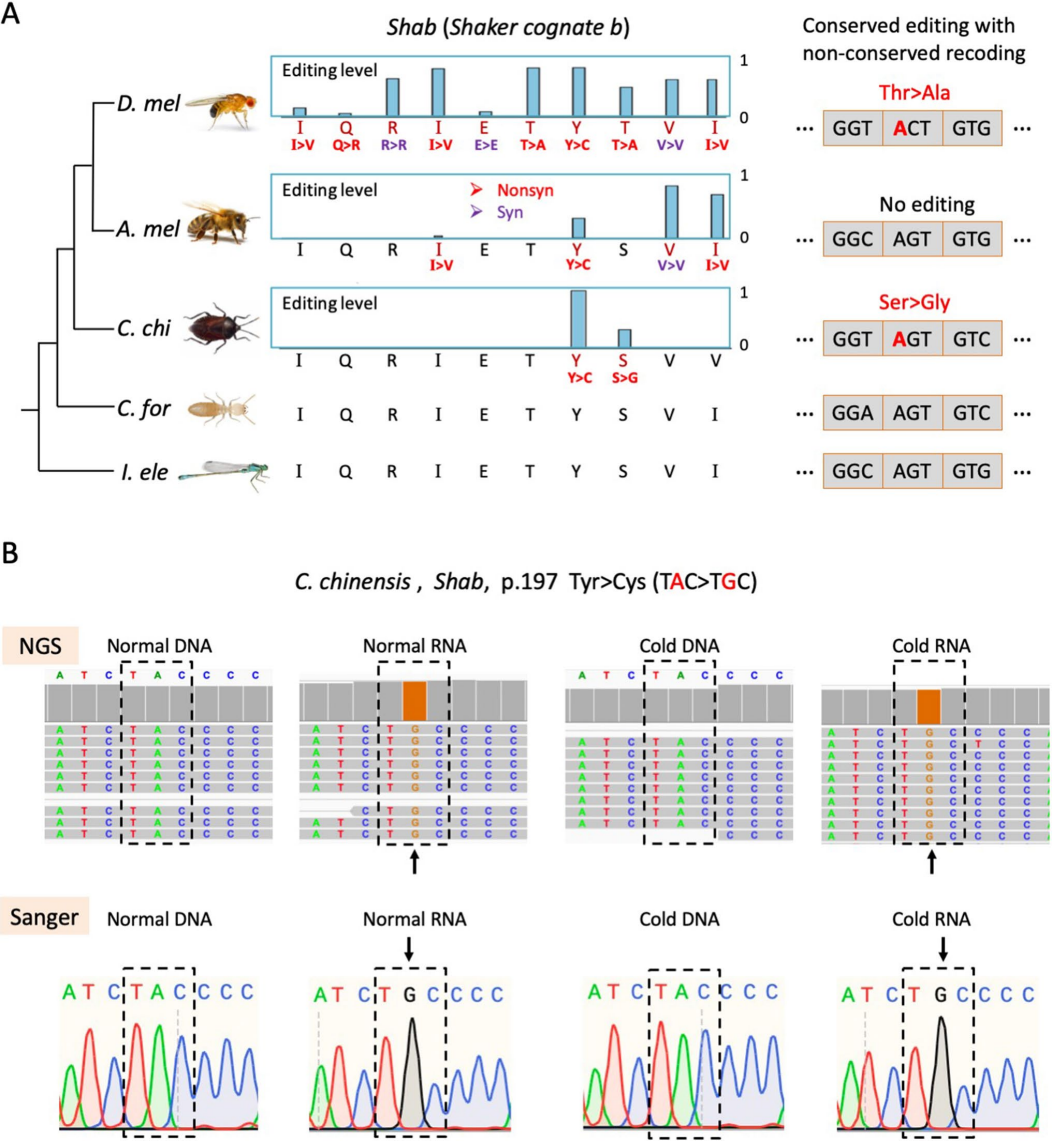
Conserved and non-conserved recoding site in gene *Shab*. **A** Genomic sequence and editing levels of editing sites in gene *Shab*. Only the editing sites and their orthologous genomic sequences were shown. Nonsynonymous editing was in red and synonymous editing was in purple. The codon context of Ser208Gly recoding in *C. chinensis* was shown as an example. The branch length of phylogenetic tree is unscaled. **B** IGV visualization and Sanger verification of Tyr197Cys recoding site in *Shab* of *C. chinensis*. DNA and RNA under normal or cold stress were displayed. Screenshots of the NGS alignments were shown for representative samples normal#4 and cold#4. The edited codon was labeled by rectangle. The male samples were shown as examples

Gene *Shab* encodes a submit of potassium channel Kv2 that regulates excitability in neurons and muscles, and governs transmitter release. We visualized the *Shab* recoding sites in *C. chinensis*, *D. melanogaster*, and *A. mellifera*, and added two insect species *Coptotermes formosanus* (Blattaria) and *Ischnura elegans* (Odonata) as outgroups to infer the ancestral state of genomic sequence on editing sites (Fig. 4A). *C. chinensis* had two editing sites in *Shab* CDS, both of which were nonsynonymous. Strikingly, the Tyr197Cys recoding site was highly conserved in insects and had nearly ~ 100% editing level in *C. chinensis* (Fig. 4A). This editing event was confirmed by manual inspection of the NGS alignments together with Sanger validation for both DNA and RNA, and the almost 100% editing level was robustly seen under normal temperature or cold stress (Fig. 4B). In heads of *D. melanogaster* and *A. mellifera*, the Tyr > Cys recoding also exists and the editing levels were 0.85 and 0.31, respectively (Fig. 4A). With the genomic sequence of two outgroup species, we inferred that the ancestral state of this Tyr > Cys site was a Tyr codon. Due to the lack of transcriptome data of the two outgroups, the adaptive nature of this Tyr > Cys recoding cannot be determined and different species might have different needs from RNA editing. If recoding is just gained before the split of Hemiptera (*C. chinensis*), then this recoding is unlikely to be restorative although *C. chinensis* has a 100% editing level. But if *C. formosanus* and *I. elegans* also have this site edited, then additional evidence is needed to understand the nature of this recoding event.

Another recoding site in *C. chinensis Shab* was the Ser208Gly site (Fig. 4A). The genomic sequence encoded Ser in honeybee and two outgroups, but not editing was observed in honeybee. In *D. melanogaster*, the site was replaced with a Thr codon and a Thr > Ala editing event was introduced (Fig. 4A). Since the transition from Ser (AGT) to Thr (ACT) only requires a single mutation at the second codon position, it demonstrates that this editing event on adenosine is highly conserved between *C. chinensis* and *D. melanogaster* although the sequence context has slightly changed, leading to “non-conserved recoding” (Fig. 4A). We defined this phenomenon as “conserved editing with non-conserved recoding”.

Notably, there was a highly edited conserved Ile > Val recoding site between *D. melanogaster*, *A. mellifera*, *Bombus terrestris*, and even cephalopods [37], and the ancestral genomes encoded Ile, but in *C. chinensis* the genome sequence was directly replaced with a Val codon (Fig. 4A). For other recoding sites observed in gene *Shab* of flies or honeybees (most of which were specific to *D. melanogaster*), the genomic sequences in *C. chinensis* and two outgroups all encoded the pre-edited AA, suggesting that the prevalent nonsynonymous sites largely recode the potassium channel Kv2.

**Fig. 5:**
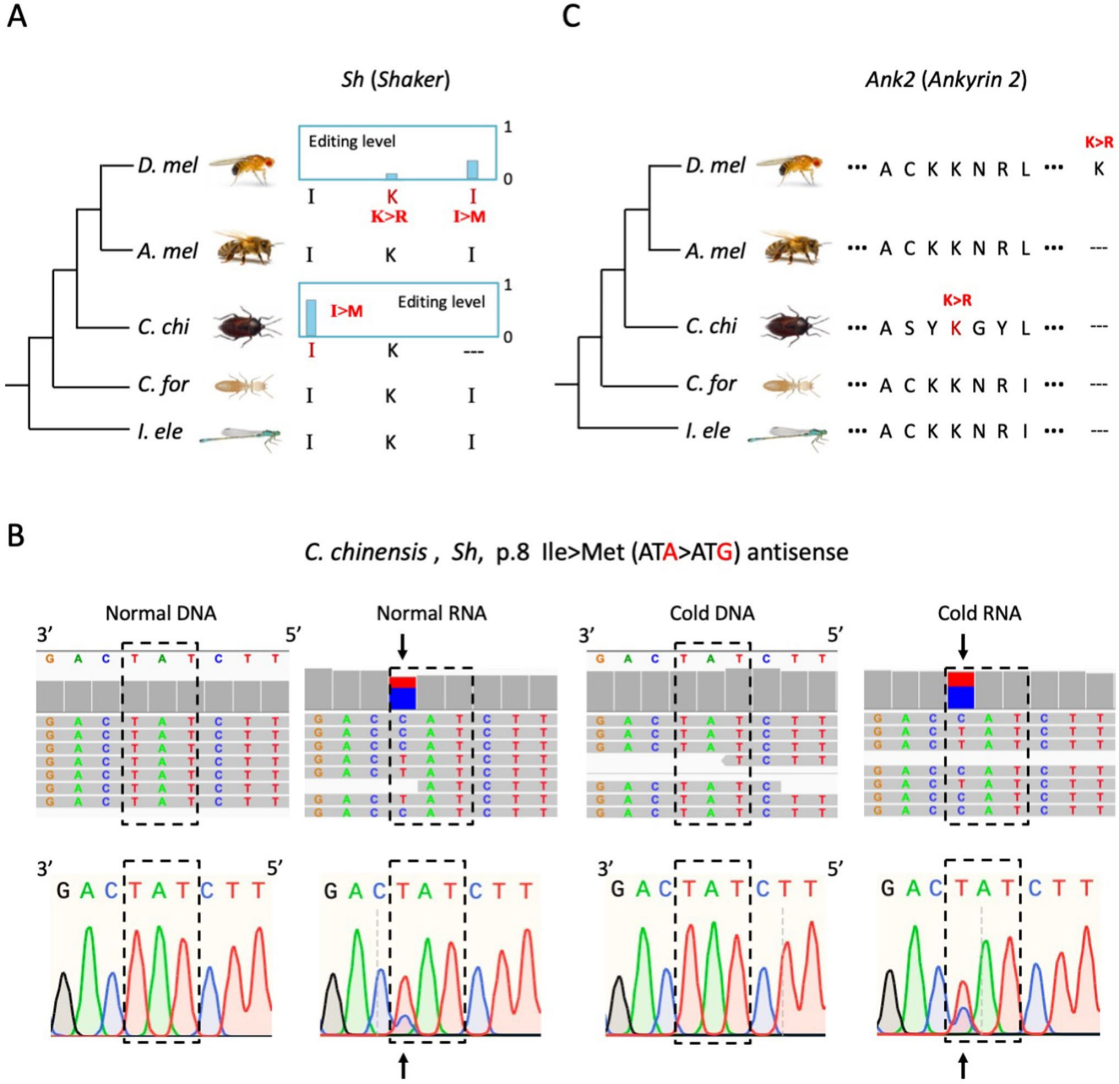
Species-specific recoding sites in genes *Sh* and *Ank2*. **A** Genomic sequence and editing levels of editing sites in gene *Sh*. Only the editing sites and their orthologous genome sequences were shown. **B** IGV visualization of Ile8Met recoding site in *Sh* of *C. chinensis*. The NGS data of DNA and RNA under normal or cold stress were displayed. Screenshots of part of the alignments were shown for representative samples normal#4 and cold#4. **C** A Lys > Arg recoding site was detected in *Ank2* gene in *C. chinensis* and *D. melanogaster*. The sequence alignment across five insect species was displayed

In addition to *Shab*, another neuronal gene with recoding in multiple species is *Sh* (*Shaker*), which also encodes a voltage-gated potassium channel (Fig. 5A). *C. chinensis* had a highly edited Ile > Met recoding site (editing level ~ 70%) and the orthologous site was all Ile in the genomes of other four species, but no editing was detected in flies or honeybees (Fig. 5A). This editing site in *C. chinensis* has been manually inspected and validated by Sanger sequencing to make sure that this recoding event was not a sequencing error or SNP (Fig. 5B). We also found two other recoding sites in *D. melanogaster* where the ancestral genome encoded the pre-edited AA (Fig. 5A). This represents the independent gains of recoding sites in neuronal genes as we previously observed between flies and bees [26].

Next, we noticed a set of paralogous genes in *C. chinensis* which all aligned to the *Ank2* (*Ankyrin 2*) gene in *D. melanogaster* (FBtr0303125). *Ank2* encodes a cytoskeletal binding protein that contributes to the regulation of short-term memory, perception, cytoskeleton and neuromuscular junction development and synapsis. This gene was recoded in both *C. chinensis* and *D. melanogaster*, but the editing sites were non-conserved. A copy of *Ank2* in *C. chinensis* (Cc07G005460.1) had a Lys > Arg recoding site mapped to a conserved genomic region (Fig. 5C), the editing level of which was lower than 0.1. Interestingly, *D. melanogaster* also had a Lys > Arg recoding site with level = 0.33, but this genomic position was deleted in all other four species (Fig. 5C). Again, this species-specific Lys > Arg recoding showed a trend of independent evolution to modify the neuron-related genes.

Since our stringent mapping, variant calling, and trimming pipelines might miss some lowly edited sites, we directly aligned the known positions of fly recoding sites to the *C. chinensis* genome and checked whether there are A-to-G events. Among the 678 recoding sites we previously identified in *D. melanogaster* brains [55], only 169 sites were adenosines in *C. chinensis* genome, and none of these sites were edited except the aforementioned examples in *Shab*. This result supports an independent evolution of the editomes seen in the two species.

### Dynamic RNA editing under cold stress of *C. chinensis*

Apart from recoding events in neuronal genes, we also found prevalent intronic editing sites. The 2783 intronic sites were located in 1165 different introns belonging to 831 unique genes. This suggests that each edited intron had on average ~ 2.4 editing sites but it was uncommon for a gene to have multiple introns edited (1.4 edited introns per gene). This could be explained by the Adar editing mode where the nearby adenosines were likely to be simultaneously edited.

Next, we start to study the effect of cold stress on the RNA editome. *C. chinensis* is able to tolerate relatively low temperature in the wild during winter, while many insects like *Drosophila* can only live on human dwellings in cold seasons. This raises an interesting question to ask whether the temperature effect would be different to the editomes of *C. chinensis* and *Drosophila*. Normally, as seen in *Drosophila*, high temperature unwinds the stable dsRNA structure and decreases the overall editing level [55]. In our *C. chinensis* data, we first looked at the global editing status under different temperatures (10°C and 26°C), and then quantitatively identified differential editing sites (DES) by pooled reads method plus the five versus five T-test (see Materials and Methods for details).

**Fig. 6:**
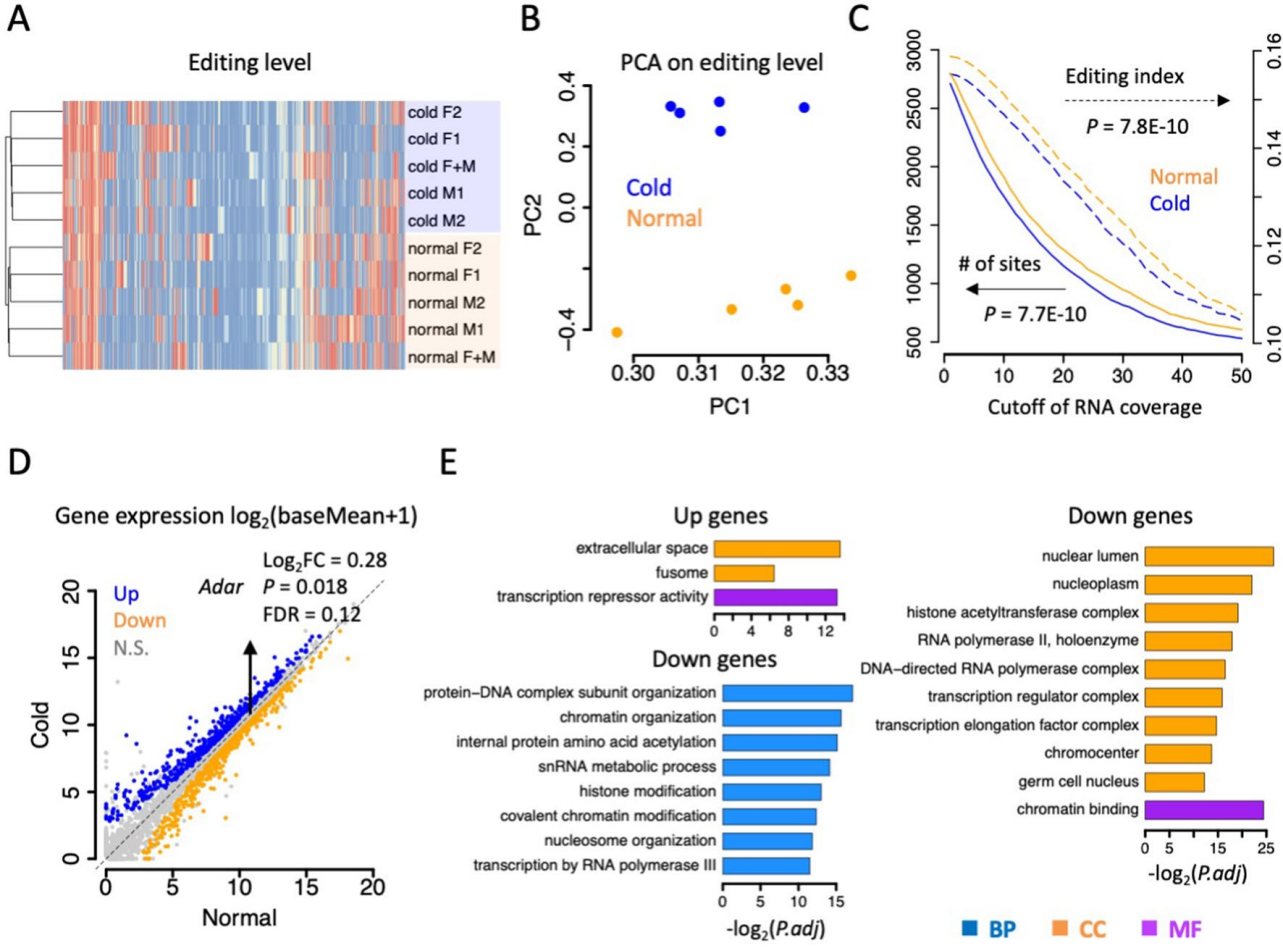
Global editing and expressional difference between normal (26℃) and cold stress (10℃). **A** Heatmap showing the clustering of editing levels across ten samples. **B** Principal component analysis (PCA) of editing levels of ten samples. Five normal samples were in orange and five cold-stressed samples were in blue. **C** Comparison of RNA editing sites and editing index under two temperatures. The reads from five samples of each condition were pooled. X-axis is the cutoff of RNA-Seq coverage for editing sites. The left axis is the number of editing sites. The right axis is editing index. *P* values were calculated by paired Wilcoxon rank sum tests. **D** Dotplots showing the gene expression of normal and cold-stressed conditions. Gene expression is represented by log_2_(baseMean + 1), where baseMean is the mean reads count normalized by DESeq2. The expression of *Adar* is slightly but not significantly up-regulated under cold stress. **E** Gene ontology of up- and down-regulated genes under cold stress. *BP* biological process, *CC* cellular component, *MF* molecular function

Hierarchical clustering of editing levels of the ten samples showed clear divergence between two temperatures, while gender effect seems negligible in shaping the editome (Fig. 6A). These patterns conform to our previous findings in *Drosophila* [55]. Moreover, principal components analysis (PCA) also revealed the distinction of the editomes under two conditions (Fig. 6B). However, while the global editing efficiency in *Drosophila* decreases with temperature due to the effect of RNA structure, the opposite trend was observed in *C. chinensis* (Fig. 6C). Under different cutoffs, the numbers of editing sites and editing indices were constantly lowers under 10°C than 26°C (Fig. 6C).

We tried to find *trans* and *cis* determinants to explain the difference in editing efficiency under different temperatures. The most intuitive connection to editing activity is the expression of Adar enzyme. To examine whether *Adar* expression explains the change in editing profile, we performed differential expression analysis to define the differentially expressed genes (DEG) under cold stress (Fig. 6D). Among the 19,701 expressed genes in *C. chinensis*, DESeq2 identified 1545 (7.8%) DEGs under FDR < 0.05, among which 700 were up-regulated and 845 were down-regulated (Fig. 6D). Interestingly, functional enrichment showed that the up-regulated genes were enriched in transcription repressor and the down-regulated genes were related to transcription and RNA polymerase (Fig. 6E). This result suggests the wide-spread shut-down of transcriptional processes under low temperature, and this strategy possibly aims to avoid unnecessary waste of energy and resources under unfavorable conditions. Notably, *Adar* (Cc04G095920) was slightly but not significantly up-regulated under cold stress (Fig. 6D), which could not explain the overall down-regulated editing efficiency. There might be other undiscovered *cis* or *trans* factors or *cis* elements that determine the editing activity. Explanations will be proposed in the Discussion section.

### Identification of differential editing sites (DES)

**Fig. 7:**
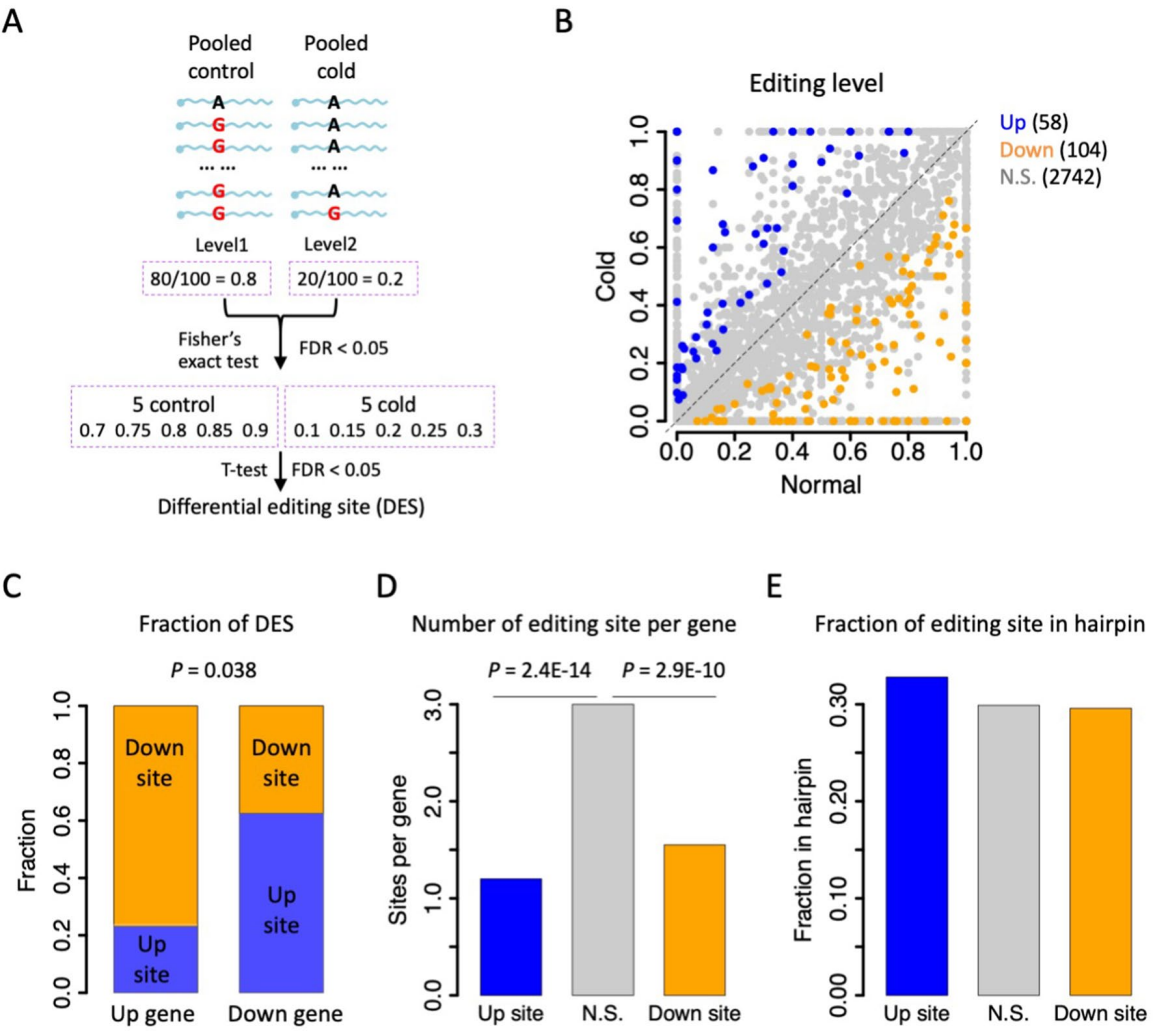
Identification of differential editing sites (DES) between normal (26℃) and cold stress (10℃). **A** A schematic diagram showing the pipeline for identifying DES. Both pooled reads strategy and five versus five tests were used to define DES. **B** Dotplot showing the pooled editing levels under two temperatures. Up-regulated sites were in blue and down-regulated sites were in orange. **C** The relative proportions of down-regulated and up-regulated sites in differentially expressed genes. *P* value was calculated by Fisher’s exact test. **D** Mean number of editing sites per gene. This number was significantly lower for DES than non-DES.* P* values were calculated by Fisher’s exact tests. **E** Fraction of editing sites located in hairpins. Structured regions with MFE < –35 were used

The overall down-regulated editing efficiency under cold stress does not imply the down-regulation of every single editing site. To get a clearer picture of the dynamic editing under different temperatures, we quantitatively defined significant differential editing sites (DES) by a series of stringent criteria combining the pooled reads method plus the five versus five T-test (see Material and Methods and Fig. 7A for details). Among the 2904 editing sites, 58 were up-regulated (55 intronic and 3 recoding sites) and 104 were down-regulated (102 intronic and two recoding sites) (Fig. 7B), while the other 2742 sites were non-DES. The fact that down-regulated sites outnumbered up-regulated sites echoed the overall lower editing efficiency under cold stress.

Interestingly, we found a tendency that up-regulated gene correlates with down-regulated editing sites and vice versa. Under the traditional DEG with FDR < 0.05, up genes possessed two up sites and seven down sites, and down genes possessed six up sites and four down sites (*P* = 0.17, Fisher’s exact test). When we expanded the DEG to FDR < 0.1, up genes would have three up sites and ten down sites, while down genes had 15 up sites and six down sites (*P* = 0.038, Fisher’s exact test, Fig. 7C). These few editing sites in DEG showed similar sequencing coverages (median depth = 20–25 for all groups of sites), and thus this robust tendency between DES and DEG did not seem to be caused by detection biases. A possible biological explanation is that higher expression means more RNA molecules produced and more substrates for the editing enzyme, but the expression level of *Adar* itself did not change significantly, making the overall editing efficiency lower. Meanwhile, we do not exclude other plausible explanations.

Moreover, when we calculated the numbers of editing sites per gene, we found that the 2742 non-DES belonged to 913 genes (3.00 sites per gene), the 104 up-regulated sites belonged to 67 genes (1.55 sites per gene) and the 58 down-regulated sites belonged to 48 genes (1.21 sites per gene). The differences between DES and non-DES were significant (Fig. 7D). This suggests that while RNA editing events tend to appear in clusters, the DESs were likely to be singletons or located far away from other sites, allowing them to be regulated separately regardless of the global effect of temperature on dsRNA and editing efficiency. Our notion was further supported by the fact that the DES and non-DES did not show differential proportions in dsRNA structures (Fig. 7E), indicating that the changes in editing levels were unlikely to be mediated by the switch in global RNA structure.

Notably, a previous study in *Drosophila* claimed that Adar seemed to be more promiscuous and less specific at higher temperature, leading to the hot-specific editing sites more disperse [88]. It echoes our result that the sites down-regulated in cold (which almost means hot-specific) were much more disperse than the overall editing sites. Moreover, the *Drosophila* work found increased hyper-editing events and decreased regular editing levels at high temperature, while in our study, the overall editing was higher under 26°C than 10°C. Since we did not distinguish hyper-editing and regular editing (as both were likely included in our results, see Materials and Methods for detailed explanation), the results from these two studies were generally compatible. Further plausible explanations will be proposed in the Discussion section. Next, we will describe representative up- or down-regulated sites in CDS or introns.

### Representative DES in CDS

**Fig. 8:**
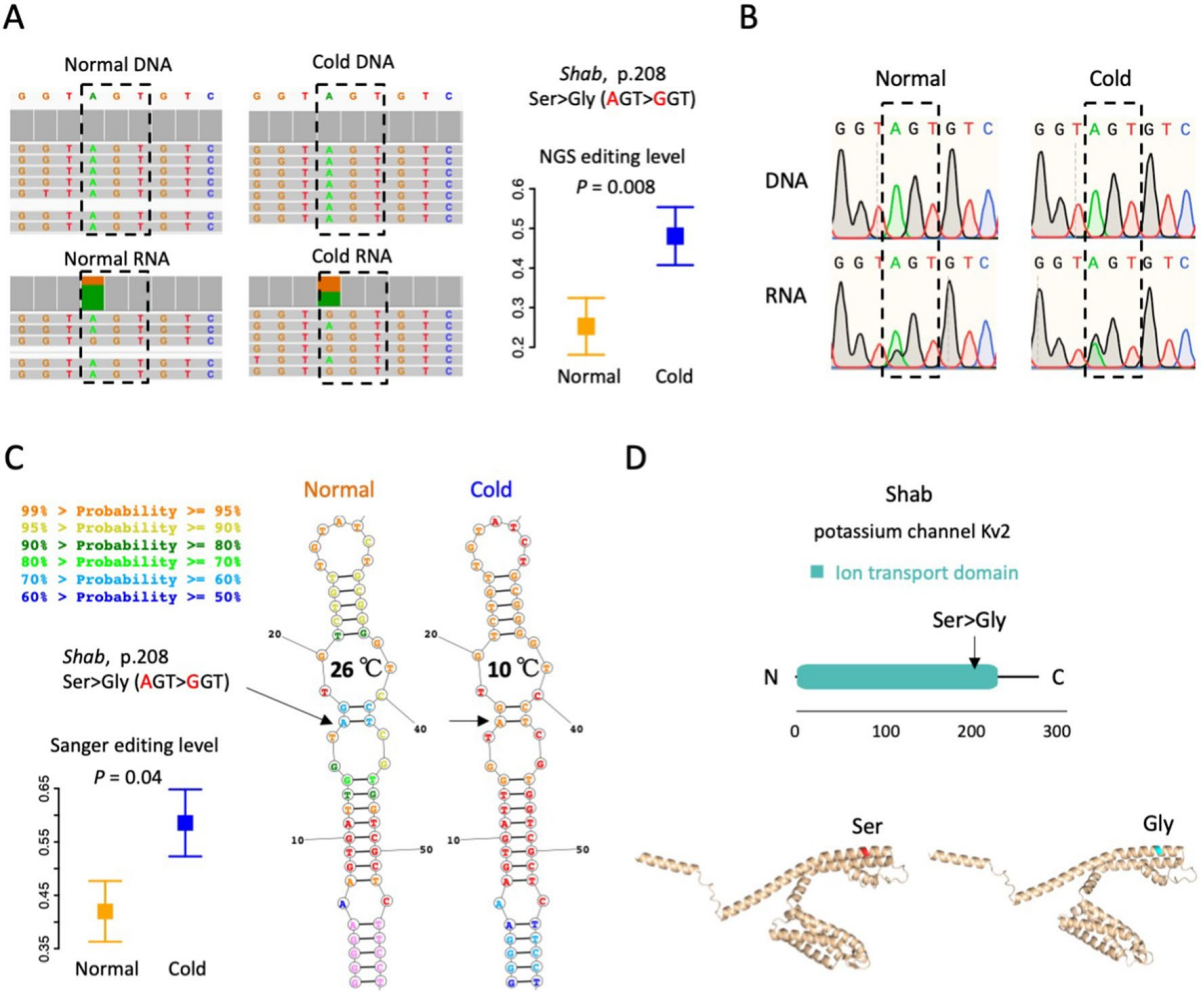
Identification of differential editing sites (DES) between normal (26℃) and cold stress (10℃). **A** Editing levels of *Shab* Ser208Gly recoding site. Error bars represent S.E. of the five samples under a temperature. The IGV visualization of representative samples normal#4 and cold#4 were displayed. **B** Sanger validation of *Shab* Ser208Gly recoding site. Both DNA and RNA were validated. Representative samples normal#4 and cold#4 were shown. The 5 versus 5 editing levels were shown in panel E. *P* value was obtained by T-test. **C** Hairpin structure around the *Shab* Ser208Gly site in pre-mRNA. The base-pairing probability of each nucleotide was indicated by color. Two temperatures were shown separately. **D** Protein architecture and AlphaFold of the protein structure of *Shab*. Structures of both the Ser and Gly versions were displayed

We first focused on the recoding DES and found that one up-regulated recoding site was the Ser208Gly site in gene *Shab*, which was, the “conserved editing with non-conserved recoding” we previously described (Fig. 4A). Gene *Shab* is highly expressed in heads, with RPKM = 10–20 across ten samples while the median RPKM values for all genes were 1–2. *Shab* expression did not show significant difference between two temperatures (*P* = 0.30 and FDR = 0.63 by DESeq2). Under 26°C, the pooled Ser > Gly editing level was 0.31 and the mean level ± S.E. for five samples was 0.25 ± 0.07, but under 10°C the pooled editing level was 0.47 and the levels for five samples were 0.48 ± 0.07 (Fig. 8A). The existence of this editing event, together with the differential levels under two temperatures, were validated by Sanger sequencing (Fig. 8B and Fig. 8C).

We noticed that this *Shab* Ser > Gly recoding site was located in a hairpin structure (Fig. 8C). Under normal temperature 26°C, the base-pairing probability of the recoding site was 60%–70%, while it was elevated to 95%–99% under 10°C (Fig. 8C). Since RNA editing largely relies on dsRNA, the increased base-pairing probability of an adenosine will enhance the Adar accessibility at this particular site, leading to more transcripts being edited at this position. Moreover, the Ser208Gly recoding site was located in the functional domain of this potassium channel (Fig. 8D) that controls excitability in neurons and muscles, it is intuitive to believe that the timely regulation of the Ser > Gly recoding level might facilitate the adaptation to cold temperature just like a similar case demonstrated in cephalopods [89, 90].

Other differentially edited recoding sites included an up-regulated Asp233Gly site in gene Cc06G023200 (tRNA splicing endonuclease subunit 2), an up-regulated Ile52Val site in gene CcunG075830 (farnesyl diphosphate synthase 2), a down-regulated His324Arg site in gene Cc06G074260 (zinc finger protein 569), and a down-regulated Lys48Arg site in gene Cc05G030090 (caspase-2-like). Their absolute editing levels and foldchanges under cold stress were not as impressive as seen in *Shab* Ser208Gly site.

### Intronic DES are enriched in DEG

Next, we investigated the 157 DES in introns including 55 up-regulated and 102 down-regulated sites. We found that 13 of such DES were also located in DEG. Interestingly, we found two down-regulated genes which had both up-regulated and down-regulated intronic sites.

**Fig. 9:**
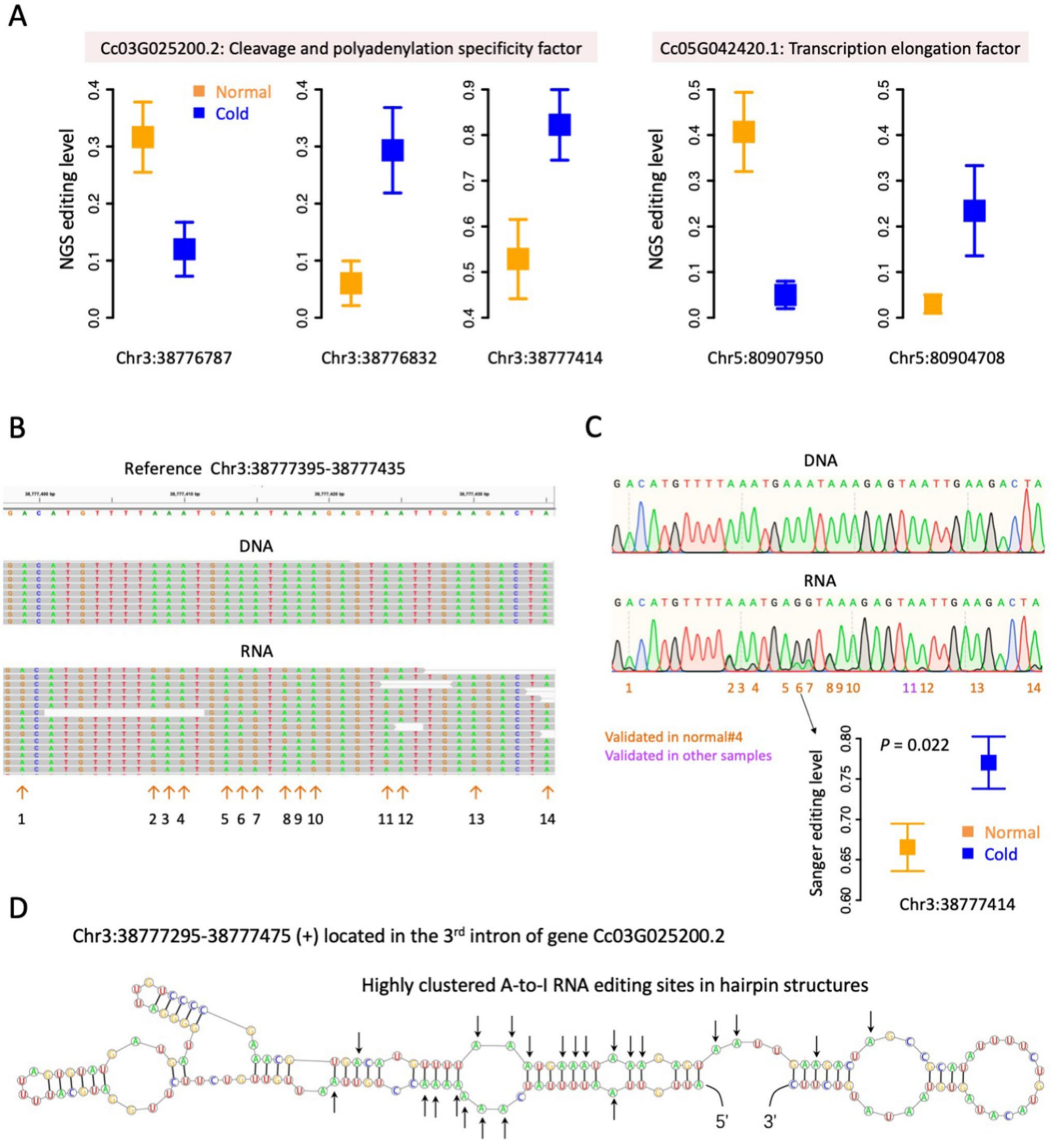
Intronic DES were enriched in DEG. **A** Editing levels of the five intronic DES in DEG. Error bars represent standard error. The genomic coordinates of each site were given below each panel. The gene names were shown above. **B** IGV visualization of 14 highly clustered editing sites in the 3rd intron of gene Cc03G025200.2. Sample normal#4 was shown. **C** Sanger validation of 14 clustered editing sites in introns. Sample normal#4 was shown. Sanger editing levels of Chr3:38,777,414 (site6) in the cluster were demonstrated. *P* value was calculated by T-test. **D** RNA structure of Chr3:38,777,295–38,777,475 in the 3rd intron of gene Cc03G025200.2. RNA editing sites were labelled

Gene Cc03G025200.2, encoding a cleavage and polyadenylation specificity factor, was significantly down-regulated under cold (log_2_foldchange = –0.31, FDR = 0.039). Site Chr3:38,776,787 located in its 3rd intron was significantly down-regulated, while sites Chr3:38,776,832 and Chr3:38,777,414 in the same (3rd) intron was significantly up-regulated (Fig. 9A). Similarly, gene Cc05G042420.1, encoding transcription elongation factor SPT4, was significantly down-regulated under cold (log_2_foldchange = –0.24, FDR = 0.024). Site Chr5:80,907,950 located in its 3rd intron was significantly down-regulated, while site Chr5:80,904,708 in the same intron was significantly up-regulated (Fig. 9A). During manual inspection of these intronic editing sites, we found a tremendous number of clustered editing sites in these regions (Fig. 9B and Supplementary Fig. S6). Particularly, 14 editing sites were detected within a 40 bp region Chr3:38,777,395–38,777,435 in the 3rd intron of gene Cc03G025200.2 (Fig. 9B). All these 14 highly clustered editing sites were validated by Sanger sequencing (Fig. 9C), and the differential editing of the focal editing site Chr3:38,777,414 was also verified (Fig. 9C). Notably, many of these 14 clustered editing sites were located in the stem of long hairpin structure (Fig. 9D). When we extended the inspected region from 40 bp to the whole hairpin, eight additional editing sites were found at upstream which were located in the opposite “strand” of the 14 editing sites (Fig. 9D). These highly clustered editing sites reflected the preference of Adar that targets nearby adenosines within a dsRNA structure.

### Validation of representative editing sites in insects under 10℃ for 30 days

Despite our interesting findings on RNA editing and gene expression patterns in *C. chinensis*, a few concerns still exist. (1) Is 10°C an extreme temperature that exceeds their cold tolerance? (2) Is 24 h of cold treatment long enough to reach an equilibrium, or 24 h would just incur an abrupt cold shock? These issues will relate to the appropriate interpretation of our observations. For the first question, we found a report on the overwintering temperature of *C. chinensis* which hibernates under 6–8℃ [91]. Moreover, the winter temperature of the place they are collected (Sichuan) is lower than 10°C. These facts suggest that 10°C is not an extremely cold temperature for the insect and thus our samples could be used for studying the transcriptomic changes under different temperatures.

Next, since we did not set up a gradual change of temperature to treat the insects, the sudden temperature change will probably incur a cold shock. We tried to answer whether 24 h of cold treatment is long enough to reach an equilibrium after the cold shock. Given the relatively long lifetime of RNA molecules at 10℃, it is possible that many of the RNAs seen after 24 h at 10℃ were actually transcribed and edited before the temperature change, and the nascent transcripts may be a small fraction of the total mRNA pool. Therefore, the differential editing sites between two temperatures might be alternatively explained by the differential degradation rates of edited and unedited transcripts. A feasible way to ease this concern is to test the alteration of editing under a prolonged set up.

Echoing the 24 h treatment of insects under 26°C and 10°C, we designed a 30 d treatment under those temperatures. Due to the limited number of individuals left, the 30 d treatment had an uneven distribution of gender (Supplementary Table S7). Seven individuals were treated under 26°C and five of them were female; ten individuals were treated under ten℃ and six of them were female. We first need to exclude the bias caused by gender. With the RNA editomes of 10 samples with 24 h treatment, we performed linear regression analysis on editing level against temperature (variable 1) and gender (variable 2). The results showed that temperature had significant contribution (*P* < 2.2E−16) to editing level while gender had no effect (*P* = 0.54). This pattern held true when we removed the two samples of mixed gender (*P* < 2.2E−16 for temperature and *P* = 0.55 for gender). We also quantitatively searched for differential editing between two genders. For each temperature, only two female and two male samples were available so that the two versus two T-test is not powerful. We therefore used Fisher’s exact test of pooled reads to identify differential editing between females and males. It turned out that none of the DES between two temperatures were differentially edited between two genders. This again supported that gender played a minor role in affecting editing level and that the unbalanced gender of 30 d treatment samples would not bias the downstream results or conclusions.

To examine whether the 24 h cold treatment could reach an equilibrium, we selected representative editing sites from the 24 h samples and carried out Sanger sequencing on the 30 d samples. Assume that the effect of cold shock is over after 30 days of 10℃ treatment, then by comparing the editing alterations in 30 d samples with those in 24 h samples, one would know whether steady state is achieved within one day, at least for the tested editing sites. The *Shab* Ser > Gly recoding level was significantly up-regulated at 24 h (Fig. 8), for the same site (Supplementary Table S8), the same tendency of increased editing levels was observed for 30 d (Supplementary Fig. S7A). Among the highly clustered editing sites in intron of gene Cc03G025200.2 (Supplementary Fig. S7B), site6 was significantly up-regulated in 24 h (Fig. 9C) and 30 d (Supplementary Fig. S8). For the down-regulated editing sites, we found that the Cc03G025200.2 intronic site8 (Supplementary Fig. S7C) and site1 (Supplementary Fig. S8) were significantly down-regulated in both 24 h and 30 d treatment, and the larger size of 30 d samples even increased the statistical power. Then, two non-DES sites were used as control: (1) *Shab* Tyr > Cys recoding site constantly had 100% editing levels in all samples tested in 24 h (Fig. 4B) and 30 d (Supplementary Fig. S7D) and thus no changes in levels were seen; (2) *Sh* Ile > Met recoding site showed no remarkably difference between two temperatures either (Supplementary Fig. S8). These Sanger validations on representative up-regulated, down-regulated, and non-DES suggest that 24 h might be enough for at least part of the editing sites to reach a steady state after the sudden cold shock.

In fact, previous studies in *Drosophila* showed that the effect of temperature on editing efficiency could be realized within 14 h, but no experiment was done to see when the steady state would be formed [55]. In cephalopods, cold-induced editing was observed within hours and reached a steady state within about 4 days [89]. These evidences support the quick adjustment of RNA editing efficiency in response to temperature change. However, since we still observed the shutdown of global transcription process in the differential expression analysis, to what extent the steady state is achieved remains to be further investigated. Importantly, with the potential effect of cold shock, we do not rule out the possibility that some differential editing sites seen at 24 h were caused by the differential stability (degradation rates) between edited and unedited transcripts. In addition, diapause (hibernation) is a major physiological change, which may have an effect on editing which is not directly related to temperature. For example, dynamic RNA editing during the hibernation was studied in heterothermic mammal squirrel, but the majority of altered sites were located in non-coding regions [92]. In our case, given that *C. chinensis* goes to diapause after 4–6 weeks of 10℃ cold treatment [93, 94] (for most insects this time is typically > 30 days [95]), our 24 h treatment is far from triggering diapause. Nevertheless, we reserve the possibility that the 30 d RNA editomes might be partially influenced by diapause.

## Discussion

Discovering the A-to-I RNA editome in new species is one of the ongoing directions of this field. High-quality characterization of new editomes will add knowledges to the evolution and adaptation of RNA editing especially when this species represents a clade without known RNA editomes. *C. chinensis* belongs to Hemiptera, the fifth largest insect order. While the other four largest insect orders are all complete metamorphosis insects and have well-characterized editomes or case studies on editing sites, Hemiptera is incomplete metamorphosis insect that diverged earlier than the other four orders, and the editome of which remains underexplored. Among the four suborders of Hemiptera, Heteroptera is the most diversified suborder, living in various habits ranging from water to terrestrial, feeding on plants, other arthropods, fungi, and animal blood [96–98]. Thus, studying the contribution of A-to-I RNA editing to the diversity and plasticity of Hemiptera/Heteroptera species is of high interest.

In this study, we assembled the chromosome-level genome of *C. chinensis* (Hemiptera: Heteroptera) and further sequenced the head transcriptomes and the matched DNA resequencing of ten *C. chinensis* samples (five 26℃ *versus* five 10℃). Like our previous findings in *Drosophila* and bee editomes [26, 55], we again found that in *C. chinensis* the nonsynonymous RNA editing events were overrepresented compared to synonymous ones. This adaptive signal suggests that overrepresented recoding exists in early-diverging insect order(s). Prevalent intronic editing was also identified. However, only very few recoding sites in well-known neuronal genes were conserved across multiple orders. For example, we found an interesting site with “conserved editing but non-conserved recoding” in potassium channel *Shab* which was significantly up-regulated in cold, serving as a candidate functional site in response to temperature stress (Fig. 4A and Fig. 8). In addition to the temperature response, this case of “conserved editing with non-conserved recoding” might suggest that the effect and function of conserved editing sites should be understood with the sequence context and amino acid information.

Under cold stress, the global editing efficiency was unexpectedly down-regulated, potentially explained by the “supply matches demand theory” upon the shut-down of general transcriptional processes (revealed by differential expression analysis). *C. chinensis* might undergo diapause under cold stress so that the overall RNA processing pathways could be down-regulated to save energies and resources, so does RNA editing. A previous study in *Drosophila* proposed that Adar became more promiscuous and less specific at higher temperature, making the hot-specific editing sites (mainly hyper-editing sites) more disperse [88]. Our results showed several similarities and also some differences to the case in *Drosophila*.

Similarity: (1) In both species, the number of total editing sites decreased under lower temperature. As we have clarified, the hyper-editing sites could be partially identified in our pipeline given a high mismatch tolerance by the aligner (see Materials and Methods), so the dynamic changes in numbers of editing sites were analogous between *Drosophila* and *C. chinensis*.

(2) In both species, the hot-specific editing sites were more disperse. In fact, since RNA editing is not an “all or none” mutation but it has a particular editing level, there is no essential difference between the condition-specific editing sites and shared editing sites in the light of differential editing. The potentially editable adenosines could all be pooled as a list of “candidate editing sites”. The so-called condition-specific sites could be regarded as the up-/down-regulated sites under a particular condition, and the extent of this “level change” at each individual site could be quantitatively measured by statistical tests. In our results, the sites significantly down-regulated under cold (suggesting hot-specific sites) were more disperse, echoing the observation in *Drosophila*.

There are also a few differences between the two species. In *Drosophila*, the overall editing index was still elevated under cold although the number of sites were fewer [88]. However, in *C. chinensis*, both editing index and the number of sites were down-regulated at lower temperature. Then, while the disperse editing sites under heat stress of *Drosophila* were explained by promiscuous Adar editing, the singleton DESs in our *C. chinensis* were believed to undergo specific regulation in response to temperature change, regardless of the global effect of temperature on RNA structure.

The difference of editome changes between *Drosophila* and *C. chinensis* might reflect their biological and physiological features. *C. chinensis* could experience diapause in wild during winter, while *Drosophila* might only live with human environments in cold seasons. Our *C. chinensis* data suggest that the up-regulated genes under cold were suppressor of transcription while the transcription-promoting genes were shut down (Fig. 6E). This expression profile suggests that the overall RNA biological processes were down-regulated under cold stress, and this strategy makes sense in the light of “supply matches demand” theory. Accordingly, RNA editing activity should be suppressed. Since the stabilized dsRNA structure at lower temperature and the insignificant difference of Adar expression did not support the decrease of global editing efficiency, it is possible that other *cis* elements or *trans* factors might exist to regulate RNA editing in *C. chinensis*.

In conclusion, ongoing efforts are paid in the identification of RNA editomes in new species, and our study provided the first RNA editome in Hemiptera that greatly advanced our understanding on the evolution, conservation, and adaptation of A-to-I RNA editing.

### Supplementary Information

Below is the link to the electronic supplementary material.

Supplementary file1 (DOCX 1320 KB)

Supplementary file2 (RAR 3081 KB)

Supplementary file3 (RAR 877 KB)

Supplementary file4 (XLSX 1035 KB)

## Data Availability

All data generated by this study was uploaded to NCBI. For genome sequencing data, the accessions numbers are SRR23604985 (RNA-Seq), SRR2360984 (Illumina short read), SRR23604986 (PacBio-HiFi read), and SRR23604983 (Hi-C data). The RNA-Seq and the DNA-Seq data for both wild control and low temperature samples were available under accession number SRP476000. The Sanger sequencing data were included in Supplementary Data 1 (24 h treatment) and Supplementary Data 2 (30 d treatment).

## Abbreviations

AA: Amino acid
A-to-I: Adenosine-to-inosine
ADAR: Adenosine deaminase acting on RNA
CDS: Coding sequence
DEG: Differentially expressed gene
DES: Differential editing sites
m^6^A: *N*^*6*^-Methyladenosine
NMD: Nonsense-mediated decay
Nonsyn: Nonsynonymous
PCA: Principal components analysis
Syn: Synonymous
S.E.M.: Standard error of mean
RSCU: Relative synonymous codon usage
CAI: Codon adaptation index
