# Supplementary material for: The first A-to-I RNA editome of hemipteran species Coridius chinensis reveals overrepresented recoding and prevalent intron editing in early-diverging insects
Source: Cell Mol Life Sci. 2024 Mar 13;81(1):136. doi: 10.1007/s00018-024-05175-6 (PMC10937787; doi:10.1007/s00018-024-05175-6)
Supplement: Supplementary file 1 — Supplementary file1 (DOCX 1320 KB) [file 18_2024_5175_MOESM1_ESM.docx]

**Supplementary Data**

**Supplementary Data 1.** Sanger sequencing raw files of the 24h treatment samples.

**Supplementary Data 2.** Sanger sequencing raw files of the 30d treatmant samples.

**Supplementary Figures**


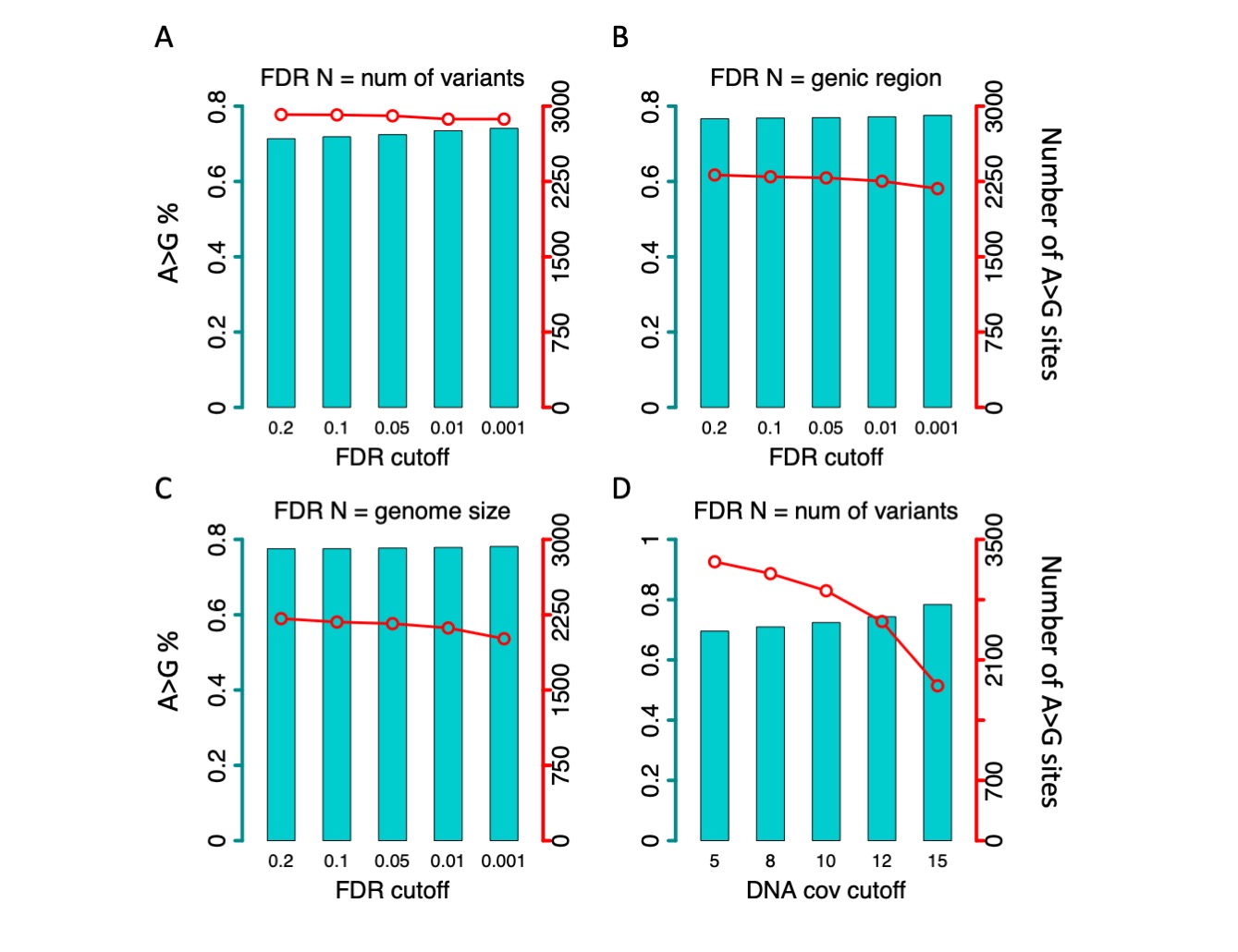


**Figure S1. Fraction of A>G variant (cyan axis) and the number of A>G sites (red axis).** (A) In FDR correction, N = default, the total number of variants. Different cutoffs on FDR were tried. (B) In FDR correction, N = the size of genic region. Different cutoffs on FDR were tried. (C) In FDR correction, N = genome size. Different cutoffs on FDR were tried. (D) In FDR correction, N = default, the total number of variants. Different cutoffs on DNA coverage were tried.


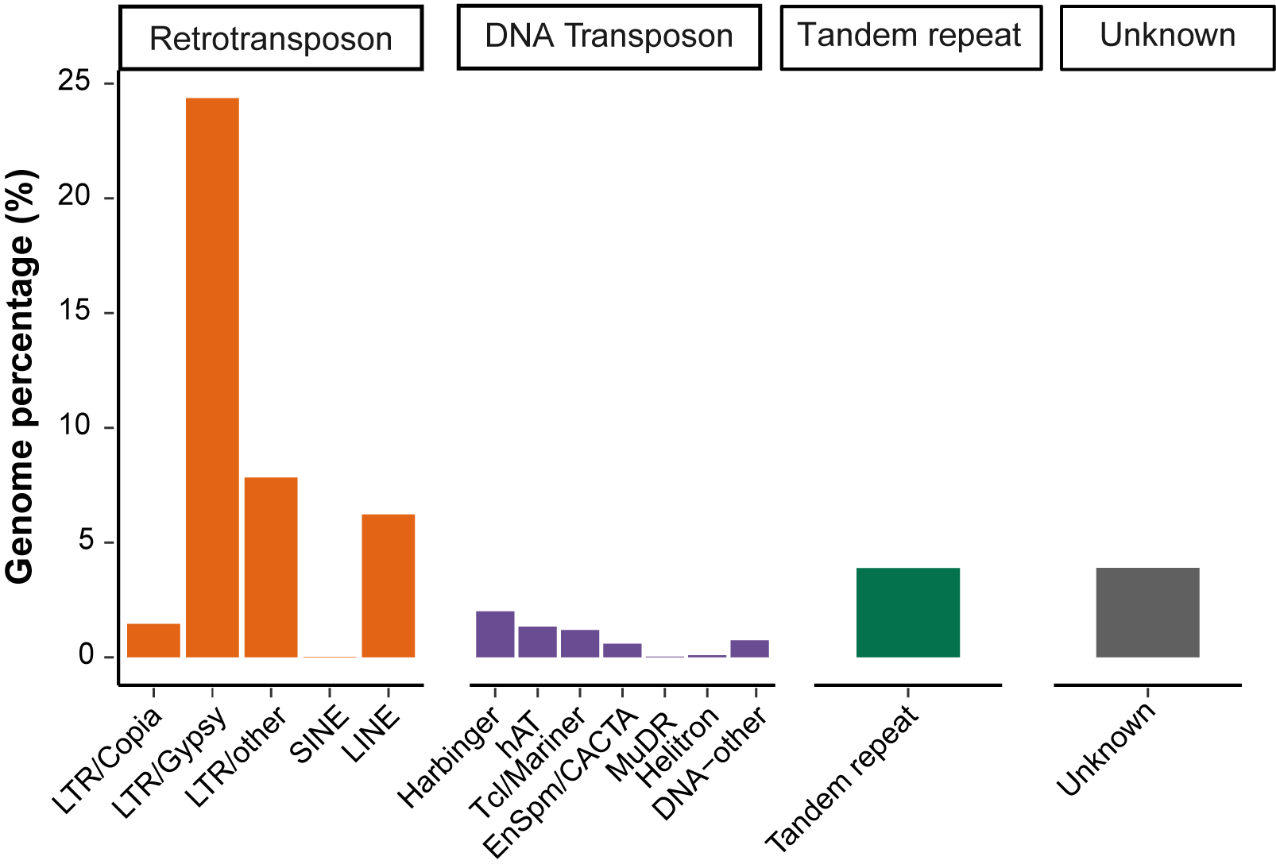


**Figure S2.** **Classification of repetative sequences and transposable elements in the genome of *Coridius chinensis*.**


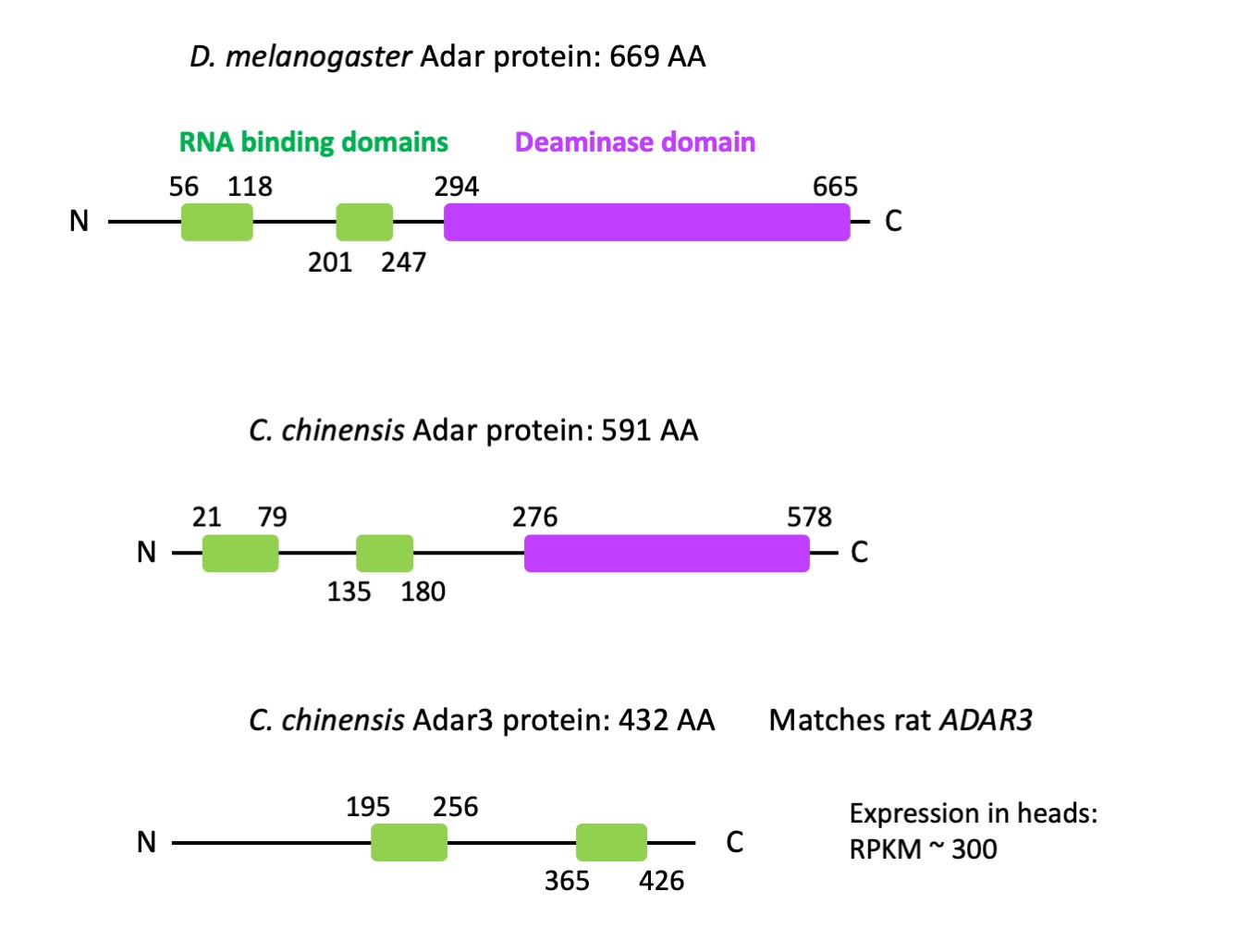


**Figure S3. Protein architectures of *Drosophila* and *C. chinensis* Adar**. The two types of domains were in different colors.


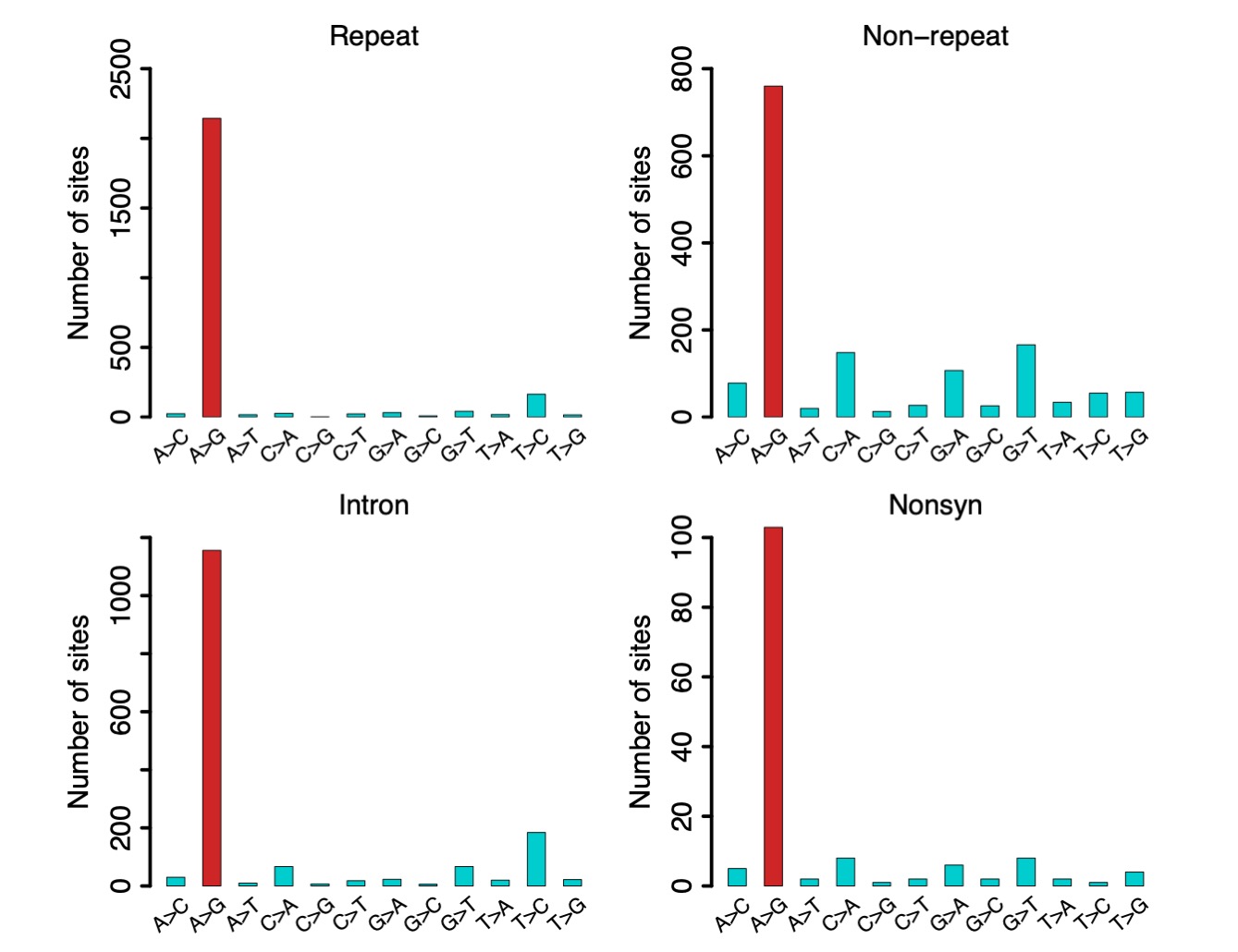


**Figure S4. Barplots showing the numbers of 12 types of RDD in genic regions.** Sites in repeat regions, non-repeat regions, introns, and nonsynonymous sites were shown respectively.


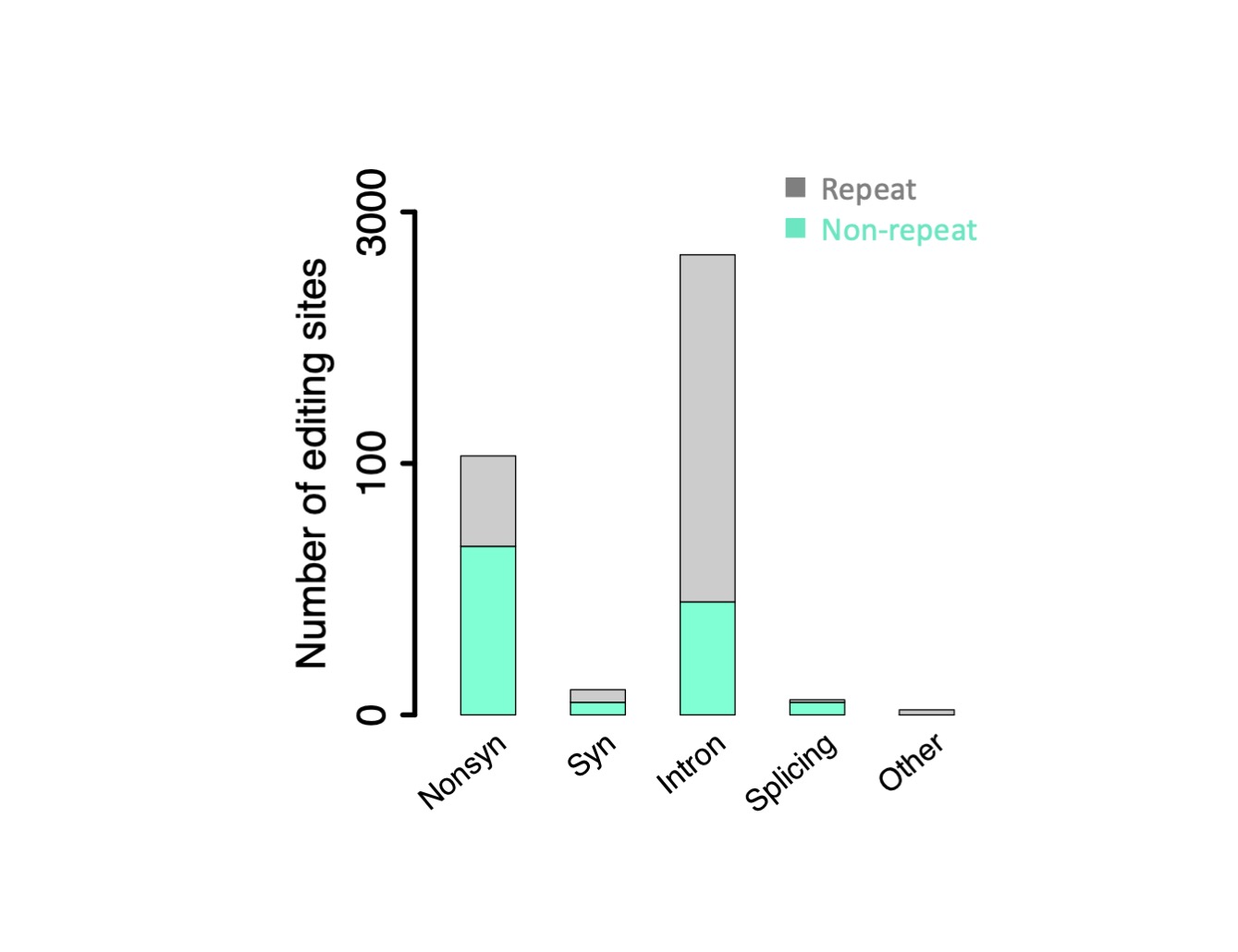


**Figure S5. Annotation of genic A-to-I RNA editing sites.** Sites in repeat and non-repeat regions were shown seperately.


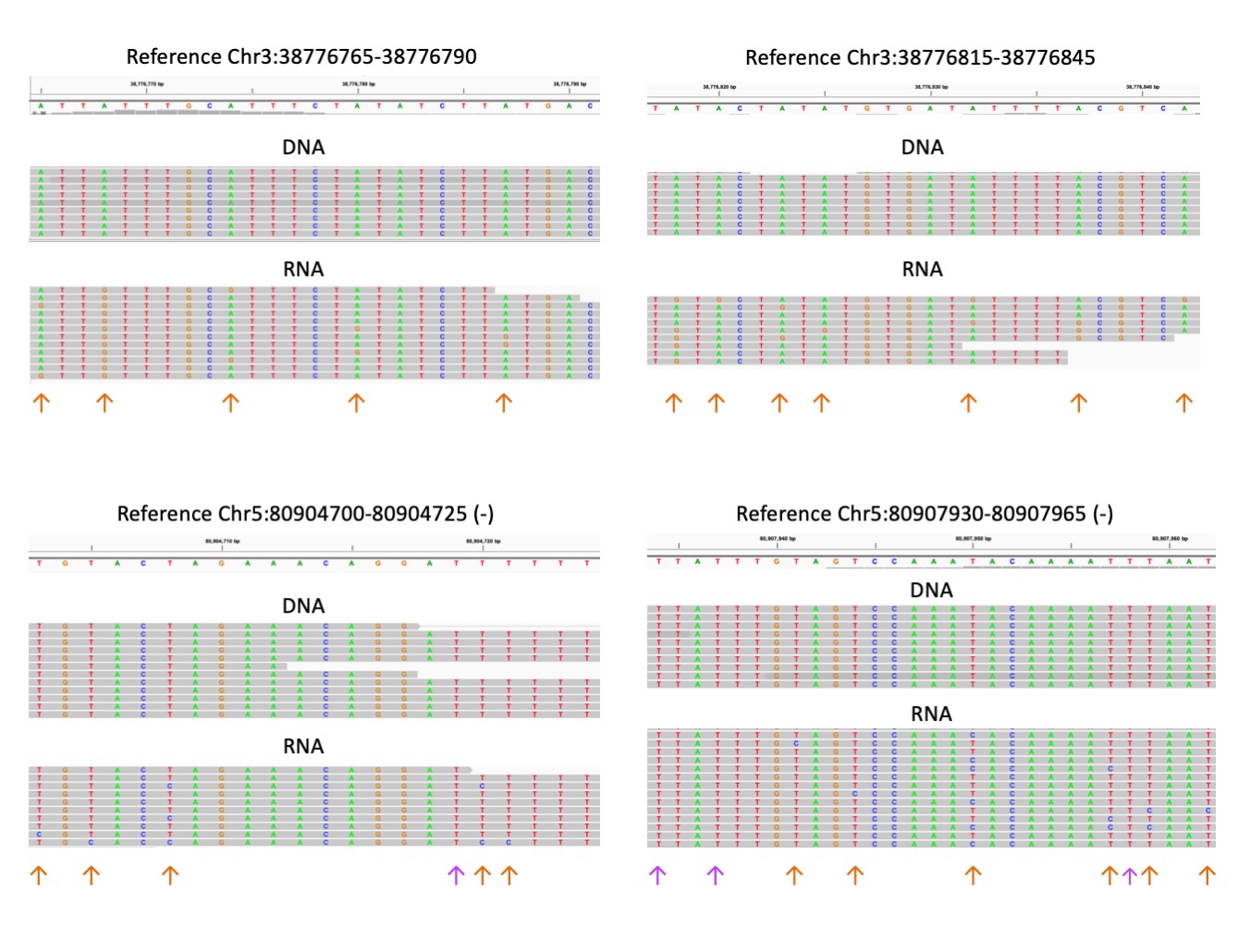


**Figure S6. Manual inspection of highly clustered editing sites via IGV.** Editing sites detected in the shown sample were in orange. Editing sites not detected in that sample but detected in other samples were in purple.


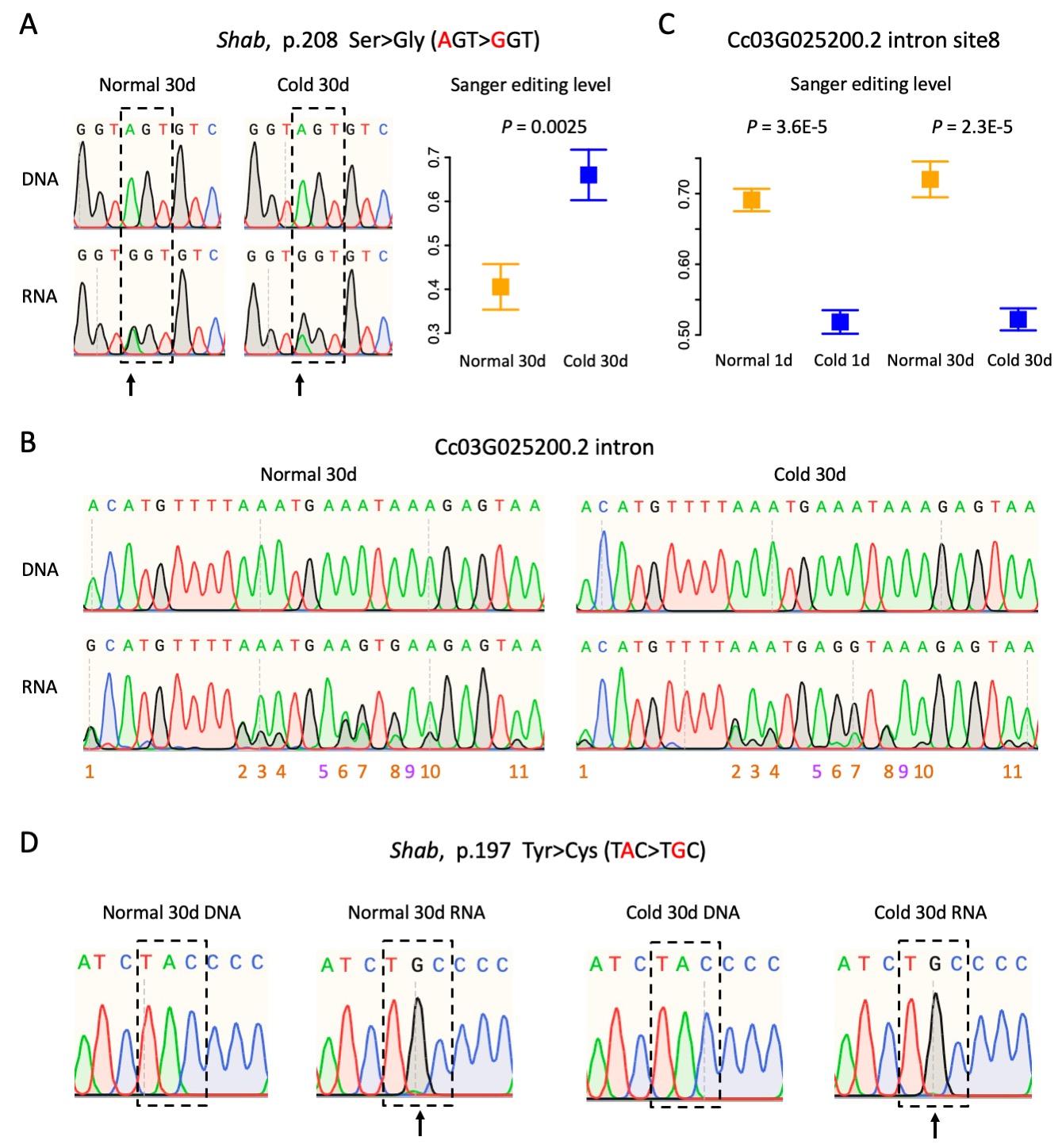


**Figure S7. Validation of representative editing sites under 10℃ for 30 days**. (A) The DNA and RNA sequences of *Shab* Ser>Gly site under normal or cold under 30 days. Sample#1 for each condition was shown. *P* value was obtained by comparing editing levels of 7 normal *versus* 10 cold samples using one-tailed T-test. (B) Sanger traces of clustered editing sites in intron of gene Cc03G025200.2. DNA and RNA sequences under normal or cold 30 days were shown. (C) Sanger editing levels of Cc03G025200.2 intronic site 8. There were 5 normal *versus* 5 cold samples treated for 24h and 7 normal *versus* 10 cold samples treated for 30 days. *P* values were obtained by one-tailed T-tests. (D) Sanger traces of *Shab* Tyr>Cys site. DNA and RNA sequences under normal or cold 30 days were displayed.


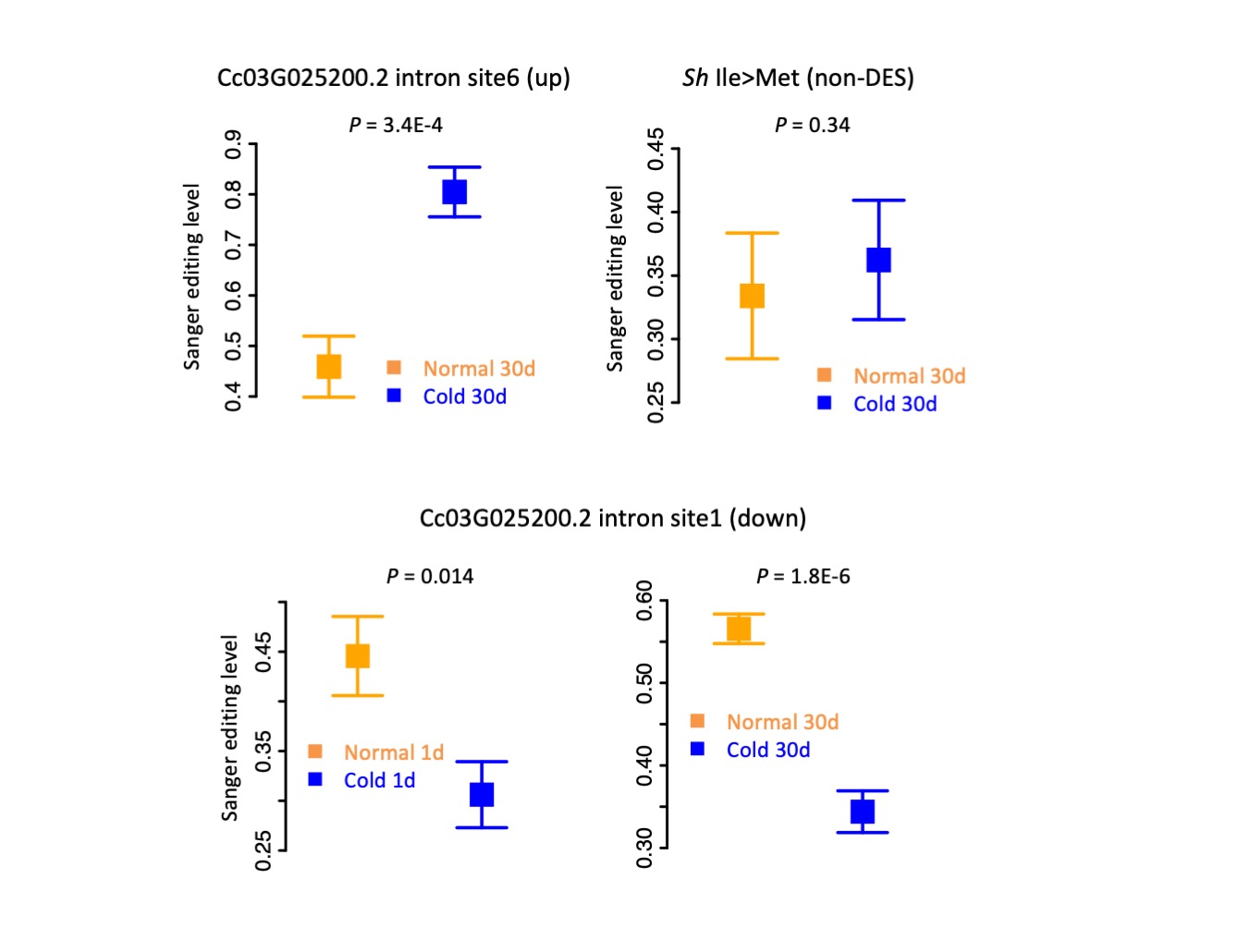


**Figure S8. Editing levels of Sanger sequencing of representative editing sites.** An up-regulated site, a down-regulated site, and a non-DES was displayed. *P* values were obtained by one-tailed T-tests.

**Supplementary Tables**

**Table S1.** PCR primers used in Sanger sequencing

| Target region | Gene | DNA/cDNA | F/R | Primer sequences |
| --- | --- | --- | --- | --- |
| Chr4:172732464 | *Shab* | Both | F | AGTTTGGCCTATTTCGCGGA |
| Chr4:172732464 | *Shab* | Both | R | GGATAGGAAGAGCGACCACG |
| Chr3:153145352 | *Sh* | DNA | F | GTGTTGGGCAGGTAAGGACA |
| Chr3:153145352 | *Sh* | DNA | R | TCTAACCTGTTCCTGGGGCT |
| Chr3:153145352 | *Sh* | cDNA | F | GCAGGTAAGGAGAAGCTTGT |
| Chr3:153145352 | *Sh* | cDNA | R | TCGGGGTGTGGGGAAA |
| Chr3:38777395-38777435 | *Cc03G025200* | Both | F | ACCTGTTAATTGTTGTCTTCTTGG |
| Chr3:38777395-38777435 | *Cc03G025200* | Both | R | AGGAAGTGACCAAGGCACAC |

F: Forward primer; R: Reverse primer.

**Table S****2.** Library and sequencing data for the *Coridius chinensis* genome assembly.

| Libraries | Insertion size | Read length (bp) | Clean data (Gb) |
| --- | --- | --- | --- |
| Illumina | 350 bp | 150 | 57.23 |
| HiFi | 20 Kb | / | 51.53 |
| Hi-C | 350 bp | 150 | 129.56 |
| RNA-seq | 350 bp | 150 | 9.72 |

**Table S3.** Summary and statistics of the *Coridius chinensis* genome assembly.

| Types | | | Contigs | Chromosomes |
| --- | --- | --- | --- | --- |
| Genome assembly | Total length (Mb) | | 1,397.7 | 1,398.3 |
|  | Max length (Mb) | | 26.8 | 283.6 |
|  | N50 length (Mb) | | 4.1 | 209.1 |
|  | N90 length (Mb) | | 0.61 | 110.1 |
|  | GC content (%) | | 33.6 | 33.6 |
| Genome assessment | BUSCO | Complete BUSCOs (%) | 94.2 | 94.4 |
|  |  | Complete and single-copy BUSCOs (%) | 93.1 | 93.4 |
|  |  | Complete and duplicated BUSCOs (%) | 1.1 | 1.0 |
|  |  | Fragmented BUSCOs (%) | 2.3 | 1.8 |
|  |  | Missing BUSCOs (%) | 3.5 | 3.8 |
|  | Quality value (QV) | | / | 33.2 |
|  | Mapping short-reads rate (%) | | / | 99.3 |

**Table S4.** Statistics of annotated genes in the *Coridius chinensis* genome.

| Features | Results |
| --- | --- |
| Gene number | 24,728 |
| Average gene length (bp) | 9,131 |
| Average CDS length (bp) | 1,022 |
| Average mRNA length (bp) | 1,241 |
| Average exon length (bp) | 311 |
| Average intron length (bp) | 2,993 |

**Table S5.** Statistics for predicted genes in the *Coridius chinensis* genome.

| Database | Annotated Number | Annotated Percent (%) |
| --- | --- | --- |
| NR | 23,359 | 94.5 |
| Swiss-Port | 16,116 | 65.2 |
| GO | 6,152 | 24.9 |
| KEGG | 9,961 | 40.3 |
| COG | 18,170 | 73.5 |
| Pfam | 15,999 | 64.7 |
| Annotated | 23,469 | 94.9 |
| Total | 24,728 | 100 |

**Table S6.** Information of identified A-to-I RNA editing sites (EXCEL)

R: reference allele count.

A: alternative allele count.

C: coverage.

L: level.

**Table S7.** Information of the individuals treated at normal (26℃) or cold (10℃) for 30 days.

| Individual ID | Gender | DNA sample ID | RNA sample ID |
| --- | --- | --- | --- |
| Cold_1 | Female | D1 | CD1 |
| Cold_2 | Female | D2 | CD2 |
| Cold_3 | Female | D3 | CD3 |
| Cold_4 | Female | D4 | CD4 |
| Cold_5 | Female | D5 | CD5 |
| Cold_6 | Male | D6 | CD6 |
| Cold_7 | Male | D7 | CD7 |
| Cold_8 | Female | D8 | CD8 |
| Cold_9 | Male | D9 | CD9 |
| Cold_10 | Male | D10 | CD10 |
| Control_1 | Female | CK1 | CCK1 |
| Control_2 | Female | CK2 | CCK2 |
| Control_3 | Female | CK3 | CCK3 |
| Control_4 | Female | CK4 | CCK4 |
| Control_5 | Male | CK5 | CCK5 |
| Control_6 | Male | CK6 | CCK6 |
| Control_7 | Female | CK7 | CCK7 |

**Table S8**. *Shab* S>G recoding level in NGS and Sanger sequencing results

| Sample | Type | Editing level |
| --- | --- | --- |
| Control_24h_1 | NGS | 0 |
| Control_24h_2 | NGS | 0.396 |
| Control_24h_3 | NGS | 0.195 |
| Control_24h_4 | NGS | 0.356 |
| Control_24h_5 | NGS | 0.316 |
| Cold_24h_1 | NGS | 0.531 |
| Cold_24h_2 | NGS | 0.632 |
| Cold_24h_3 | NGS | 0.435 |
| Cold_24h_4 | NGS | 0.220 |
| Cold_24h_5 | NGS | 0.585 |
| Control_24h_1 | Sanger | 0.314 |
| Control_24h_2 | Sanger | 0.321 |
| Control_24h_3 | Sanger | 0.626 |
| Control_24h_4 | Sanger | 0.442 |
| Control_24h_5 | Sanger | 0.397 |
| Cold_24h_1 | Sanger | 0.758 |
| Cold_24h_2 | Sanger | 0.462 |
| Cold_24h_3 | Sanger | 0.558 |
| Cold_24h_4 | Sanger | 0.702 |
| Cold_24h_5 | Sanger | 0.448 |
| Control_30d_1 | Sanger | 0.462 |
| Control_30d_2 | Sanger | 0.505 |
| Control_30d_3 | Sanger | 0.472 |
| Control_30d_4 | Sanger | 0.498 |
| Control_30d_5 | Sanger | 0.482 |
| Control_30d_6 | Sanger | 0.252 |
| Control_30d_7 | Sanger | 0.167 |
| Cold_30d_1 | Sanger | 0.706 |
| Cold_30d_2 | Sanger | 0.802 |
| Cold_30d_3 | Sanger | 0.768 |
| Cold_30d_4 | Sanger | 0.718 |
| Cold_30d_5 | Sanger | 0.903 |
| Cold_30d_6 | Sanger | 0.646 |
| Cold_30d_7 | Sanger | 0.299 |
| Cold_30d_8 | Sanger | 0.690 |
| Cold_30d_9 | Sanger | 0.671 |
| Cold_30d_10 | Sanger | 0.398 |
